# Supplementary material for: Adherence to voluntary UK sugar, salt, and calorie reduction targets in the highest-grossing restaurant chains: A cross-sectional study
Source: PLoS Med. 2026 May 5;23(5):e1004681. doi: 10.1371/journal.pmed.1004681 (PMC13143115; doi:10.1371/journal.pmed.1004681)
Supplement: S1 File — (PDF) [file pmed.1004681.s001.pdf]

Assessing adherence to Government's sugar, salt, and calorie reduction targets of the top 20 UK restaurants' menus in 2024: A cross-sectional study

Alice O'Hagan, Hannah Forde, Joseph Rosenfeld, Rosie Cooper, Rachel Pechey and Lauren Bandy

Background

The purchasing and consumption of foods high in energy, saturated fat, free sugars and/or salt is an important marker of poor diet, and is associated with an increased risk of obesity and diet-related non-communicable disease (1). Over the last decade, UK Government have implemented various programmes centred around encouraging the food industry to address this public health concern. One such programme involved publishing sets of voluntary guidelines that retailers, manufacturers, and the out-of-home sector could follow in order to reduce the sugar, salt, and calorie content of their food products. The first of such guidelines was published in 2004 and recommended a reduction in daily salt intake to 6g for adults and lower for children (2). Since this release there have been numerous revisions to the guidelines, with the most recent being published in 2020 (3). Complementary to the salt reduction scheme, additional targets were set for sugar (4) and calories (5), with the goal that the sugar targets were met by 2020 and the salt and calorie targets by 2024. Adherence to these guidelines was on a voluntary basis with no direct enforcement by Government, despite similar voluntary schemes showing little engagement from the food industry and little impact on public health as a result (6). Furthermore, voluntary regulations have been shown to be less effective than mandatory regulations in encouraging food and drink companies to change their practices (7).

A considerable proportion of UK individuals' weekly food intake comes from meals prepared out-of-home such as from takeaways or restaurants (8), making the nutritional quality of this food a key determinant of the population's health. Eating out-of-home is becoming more popular, with a 159% increase in eating out expenditure from 2021 to 2022 in the UK (9). This growth in popularity may be due to the comparatively lower cost and higher convenience of eating out-of-home compared to eating at home (10). The out-of-home sector is dominated by a small number of multinational companies who operate chained food outlets, including McDonald's, Dominos, KFC, Burger King, and Subway. In 2023, sales from chained food service outlets such as these reached £35.1 billion in the UK and has increased by nearly £8 billion over the last two years (11). The growing popularity of the out-of-home sector increases the influence of such companies on the population's diet

quality and subsequent health outcomes, which highlights the need for monitoring within this industry.

Governmental progress reports of the sugar, salt, and calorie reduction targets indicate very little engagement from the out-of-home sector overall. A negligible reduction of 0.2% was observed in the average total sugar per 100g for the out-of-home sector between 2017 and 2020 (12). More promising results were seen on the food-category level with cakes and morning goods showing an 8.2% and 3.5% reduction respectively, whilst the other three included food categories showed small increases of 0.5% at most (12), although these were all far off the 20% target. The calorie targets have shown comparatively less progress, with small increases (up to 2.3%) being seen in the number of calories in main meals, starters and side dishes, and sandwiches (13). The salt targets paint a similar picture, with a decrease from 74% to 61% in the percentage of products falling below the maximum salt content targets from 2017 to 2018 (limited to products with sufficient nutritional data) (14). There is some evidence from the academic literature for reductions in sugar and salt content, namely a 5.2% decrease in sugar content from 2015 to 2018 (15) and a 15% reduction in urinary sodium since the introduction of the salt targets (16), but seemingly no assessment of the calorie targets to date.

Currently there is less evidence that documents changes in sugar, salt, or calories occurring at the company level, partly because data about the out-of-home sector is less readily available. One study that did consider company-level changes (only in the retail sector) found that of the top 10 companies across 5 target food categories, just under half met the 5% sugar reduction targets for 2018 (15), highlighting the variability in companies' adherence to the targets. As the lack of Government enforcement places the responsibility onto food companies, understanding the nutrient content changes happening within individual companies would improve transparency about their commitment to public health, and potential variation between companies. Knowing which companies are performing better or worse against the Government targets could help with enforcement of comparable targets in the future, and underpin other approaches to incentivising healthier food provision (e.g., investment decision-making based on food healthiness).

Aims

This study primarily aims to assess the nutritional content (sugar, salt, and calories) of food and drink items sold in 2024 by the top 20 highest-grossing chained restaurants in the UK (highest-grossing in 2022). Secondary to this, we will assess the UK FSA/Ofcom nutrient profile model (NPM) score (17) of these items and changes in their nutritional content between 2022 and 2024.

Each product's sugar, salt, and calorie content will be compared against the most recent reduction targets (2020 for sugar and 2024 for salt and calories) and the proportion of each companies' products that meet the targets will be calculated. The UK FSA/Ofcom NPM will

also be applied, assigning each product as 'unhealthy' or 'healthier' based on their nutritional information, and the proportion of each companies' products that are classified as 'healthier' will be calculated. A comparison will be conducted between 2022 and 2024 product data from the same 20 companies, to explore any longitudinal changes in the nutrient content or healthiness of products.

Methods

Data Sources

Data from market research company Euromonitor International will be used to identify the top 20 chained restaurants in the UK in 2022. A company will be excluded if it does not provide full nutrition information online or if the menu is different for each individual restaurant branch, such as in a chain of pub restaurants with site-level management.

The data will be collected by hand directly from the restaurant website and imputed into Excel. The data that will be collected where available are: product name, restaurant/company name, nutrient content (kcal, carbohydrate, fat, protein, fibre and salt) per 100g and per serving size, and serving size values in grams. A product will be excluded if it does not have information for at least one of the nutrients of interest (sugar, salt, or calories).

We will report the date of data collection, whether the data was extracted from a webpage or PDF resource, and whether any nutrient content values were not reported (example Table 1).

Table 1: Descriptive statistics

**Restaurant name**	Number of menu items (n)	Nutrition info given per 100g (Y/N)	Nutrition info given per serving size (Y/N)	Serving sizes given (Y/N)	Menu type/extraction method (online, PDF)	Any missing nutrient info?

2022 and 2024 were selected as the data collection periods for this study as other government policies have considered a two-year window to be a sufficient time period for

food and drinks companies to change their product portfolio (for example, the Soft Drinks Industry Levy which was announced in 2016 and implemented in 2018 (18)).

Analysis

The analysis will be conducted in Excel and R.

1. Categorisation. Each product will be assigned an overall category (e.g. main meals, sides, desserts, drinks) based on the way they are described in the menus, as well as the categories outlined by the calorie, sugar and salt reduction targets.
2. Serving size and per 100g calculations. If per 100g data is not given, then serving size data will be used to calculate data per 100g. Where serving size is not given, average serving sizes per category will be calculated using data where both serving size and per 100g is given. If this is insufficient, then serving sizes values from the Food Standards Agency “Food Portion Sizes” handbook (19) will be used.
3. The UK Ofcom/FSA nutrient profile model will be applied to per 100g data to score products by restaurant and by category. “A points” are awarded for a product’s energy, saturated fat, sugar and salt content per 100g, and “C points” are awarded for fibre, protein and fruit nut and vegetable content. Foods that score 4 points and drinks that score 1 point or more are more are classified as ‘less healthy’. For the purpose of comparing companies’ entire product portfolios, the nutrient profile score will be converted to a 1-100 scale (-2*(original score) +70), so that a higher score indicates a healthier product.
4. The nutrition information for each restaurant and category will also be compared to the calorie, salt, and sugar reduction targets for the out of home sector.
5. Products will be matched between the 2022 and 2024 datasets based on their name and company affiliation, and compared in their nutritional content, NPM classification, and adherence to the targets.

Outcomes and Statistical Analyses

Primary Outcomes

- The mean sugar, salt, and energy (kcal) content of menu items by restaurant and category.
- The proportion of menu items that meet the sugar, salt, and calorie targets by restaurant and category.
- The proportion of menu items that meet all applicable targets (not all products have targets for all three nutrients) by restaurant and category.

Table 2 and 3 provide examples of how the primary outcomes will be presented, which will be supplemented by bar charts displaying the proportional or mean nutrient information by restaurant and category.

Table 2: Mean nutrient content and % of menu items meeting targets, by restaurant

**Restaurant name**	Mean calorie content	% items meeting calorie targets	Mean sugar content	% items meeting sugar targets	Mean salt content	% items meeting salt targets	% items meeting all applicable targets

Table 3: Mean nutrient content and % of menu items meeting targets, by category

**Food category**	Mean calorie content	% items meeting calorie targets	Mean sugar content	% items meeting sugar targets	Mean salt content	% items meeting salt targets	% items meeting all applicable targets

Primary Analyses

Factorial ANOVAs will be conducted with the independent variables of restaurant and category, and dependent variable of mean nutrient content per 100g (separate ANOVAs for sugar, salt, and kcal).

Logistic regressions will be conducted with the predictor variables of restaurant and category, and response variable of whether or not a product meets its sugar, salt, calorie, and all applicable targets (separate regressions for sugar, salt, calorie, and all applicable targets).

Sensitivity analyses

The primary analyses will be re-run with:

- ‘Limited time menu’ products excluded from the dataset.
- The ‘per serving’ nutritional information to reflect the product as sold rather than following the companies’ recommended serving size.
- Products for which the calculated mean portion size of a category was used (due to actual portion size not being reported), the FSA standardised portion size to be used instead.

Secondary Outcomes

- Mean NPM score by restaurant and category.
- The proportion of products classified as ‘healthier’ by restaurant and category.

Bar charts will be used to illustrate the proportion of healthier products by restaurant and category.

Secondary Analyses

A Factorial ANOVA will be conducted with the independent variables of restaurant and category, and dependent variable of mean NPM score.

A Logistic regression will be conducted with the predictor variables of restaurant and category, and response variable of whether or not a product has a ‘healthier’ NPM classification.

Exploratory Outcomes

Products will be matched between the 2022 and 2024 datasets based on exact matches with restaurant name and product name.

Clustered bar charts will be used to illustrate:

- The proportion of products classified as healthy in 2022 and 2024, by restaurant and category.
- The proportion of products that meet the sugar, salt, and calorie targets in 2022 and 2024, by restaurant and category.

- The proportion of products that meet all applicable targets in 2022 and 2024, by restaurant and category.

Exploratory Analyses

Factorial ANOVAs will be conducted with the independent variables of restaurant and category, and dependent variable of 'change in nutrient content from 2022' (separate ANOVAs for sugar, salt, and kcal).

McNemar tests will be conducted with the grouping variable of time (2022 or 2024) and outcome variable of proportion of products that met their targets (separate tests for sugar, salt, calorie, and all applicable targets).

References

1. Thapsuwan, S., Phulkerd, S., Chamratrithirong, A., Gray, R.S., Jindarattanaporn, N., Loyfah, N., Thongcharoenchupong, N. and Pattaravanich, U., 2024. Relationship between consumption of high fat, sugar or sodium (HFSS) food and obesity and non-communicable diseases. *BMJ Nutrition, Prevention & Health*, p.e000794.
2. GOV.UK [Internet]. [cited 2024 May 08]. Voluntary – Salt reduction Strategy Reformulation Targets. Available from: 10-1283-voluntary-salt-reduction.pdf (publishing.service.gov.uk)
3. GOV.UK [Internet]. [cited 2024 May 08]. Salt reduction targets for 2024. Available from: 2024_salt_reduction_targets_070920-FINAL-1.pdf (publishing.service.gov.uk)
4. GOV.UK [Internet]. [cited 2024 May 08]. Sugar reduction: the evidence for action. Available from: Sugar_reduction_The_evidence_for_action.pdf (publishing.service.gov.uk)
5. GOV.UK [Internet]. [cited 2024 May 08]. Calorie reduction: the scope and ambition for action. Available from: Calories_Evidence_Document.pdf (publishing.service.gov.uk)
6. Panjwani, C. and Caraher, M., 2014. The Public Health Responsibility Deal: brokering a deal for public health, but on whose terms?. *Health Policy*, *114*(2-3), pp.163-173.
7. Galbraith-Emami, S. and Lobstein, T., 2013. The impact of initiatives to limit the advertising of food and beverage products to children: a systematic review. *Obesity reviews*, *14*(12), pp.960-974.
8. Adams, J., Goffe, L., Brown, T., Lake, A.A., Summerbell, C., White, M., Wrieden, W. and Adamson, A.J., 2015. Frequency and socio-demographic correlates of eating meals out and take-away meals at home: cross-sectional analysis of the UK national diet and nutrition survey, waves 1–4 (2008–12). *International Journal of Behavioral Nutrition and Physical Activity*, *12*, pp.1-9.
9. GOV.UK [Internet]. [cited 2024 May 08]. Family Food FYE 2022. Available from: Family Food FYE 2022 - GOV.UK (www.gov.uk)
10. Gesteiro, E., García-Carro, A., Aparicio-Ugarriza, R. and González-Gross, M., 2022. Eating out of home: influence on nutrition, health, and policies: a scoping review. *Nutrients*, *14*(6), p.1265.
11. Passport GMID 2024. "Consumer food service by type, market sizes and brand shares". Accessed online via the Bodleian Library.
12. GOV.UK [Internet]. [cited 2024 May 08]. Sugar reduction programme: report on industry progress 2015 to 2020. Available from: Sugar-reduction-and-reformulation-progress-report-2015-to-2020.pdf (publishing.service.gov.uk)
13. GOV.UK [Internet]. [cited 2024 May 08]. Calorie reduction programme: industry progress 2017 to 2021. Available from: Calorie reduction programme: industry progress 2017 to 2021 - GOV.UK (www.gov.uk)
14. GOV.UK [Internet]. [cited 2024 May 08]. Report: the food industry's progress towards meeting the 2017 salt targets. Available from: Salt_targets_2017_Second_progress_report_031020.pdf (publishing.service.gov.uk)
15. Bandy LK, Scarborough P, Harrington RA, Rayner M, Jebb SA. The sugar content of foods in the UK by category and company: A repeated cross-sectional study, 2015-2018. PLOS Med. 2021 May 18;18(5):e1003647.

16. He FJ, Brinsden HC, MacGregor GA. Salt reduction in the United Kingdom: a successful experiment in public health. J Hum Hypertens. 2014 Jun;28(6):345–52.
17. GOV.UK [Internet]. [cited 2024 May 08]. Nutrient Profiling Technical Guidance. Available from: dh_123492.pdf (publishing.service.gov.uk)
18. GOV.UK [Internet]. [cited 2024 May 13]. Soft Drinks Industry Levy. Available from: Soft Drinks Industry Levy - GOV.UK (www.gov.uk)
19. Food Standards Agency (2002) *Food Portion Sizes*. London: The Stationary Office.
