# Supplementary material for: Adherence to voluntary UK sugar, salt, and calorie reduction targets in the highest-grossing restaurant chains: A cross-sectional study
Source: PLoS Med. 2026 May 5;23(5):e1004681. doi: 10.1371/journal.pmed.1004681 (PMC13143115; doi:10.1371/journal.pmed.1004681)
Supplement: S1 Table — The Strengthening the Reporting of Observational Studies in Epidemiology (STROBE) Statement: Guidelines for Reporting Observational Studies von Elm E, Altman DG, Egger M, Pocock SJ, Gøtzsche PC, et al. (2007) The Strengthening the Reporting of Observational Studies in Epidemiology (STROBE) Statement: Guidelines for Reporting Observational Studies. PLOS Medicine 4(10): e296. https://doi.org/10.1371/journal.pmed.0040296. (PDF) [file pmed.1004681.s002.pdf]

**S1 Table. STROBE Checklist**

| | Item No. | Recommendation | Respected? | Comments and quotes |
|---|---|---|---|---|
| **Title and abstract** | 1 | (*a*) Indicate the study's design with a commonly used term in the title or the abstract | Yes | Study design is indicated in the title with the phrase 'A Cross-sectional Study'. |
| | | (*b*) Provide in the abstract an informative and balanced summary of what was done and what was found | Yes | The abstract includes a 'Methods/Findings' section which provides information on our study approach and findings. |
| **Introduction** | | | | |
| Background/rationale | 2 | Explain the scientific background and rationale for the investigation being reported | Yes | The Background section outlines the previous literature evaluating the UK Government's sugar, salt, and calorie reduction targets, followed up by how this study will address the outstanding gaps in this literature. |
| Objectives | 3 | State specific objectives, including any prespecified hypotheses | Yes | The aim of this study is described in the final paragraph of the Background section: "This study aimed to assess the nutritional content and adherence to the UK Government's sugar, salt, and calorie reduction targets for food items offered by the highest grossing restaurant chains in the UK, in 2024.". No specific hypotheses were specified. |
| **Methods** | | | | |
| Study design | 4 | Present key elements of study design early in the paper | Yes | A summary of our methodology approach is given in the first paragraph of the Methods section. |
| Setting | 5 | Describe the setting, locations, and relevant dates, including periods of recruitment, exposure, follow-up, and data collection | Yes | As our study used nutritional data, there is no setting or location of the study. We do however include the months in which data collection took place (subheading two of the Methods section, 'Data |

| | | | | |
|---|---|---|---|---|
| | | | | Collection'): "Menu data was collected directly from restaurants' UK websites in February and March of 2024 (May 2024 for Subway)." |
| Participants | 6 | (*a*) Give the eligibility criteria, and the sources and methods of selection of participants | Yes | The 'Identifying Restaurants' section (first subheading of the Methods) outlines how we selected the restaurants to include in our sample, and the criteria restaurants had to meet in order to be included. |
| Variables | 7 | Clearly define all outcomes, exposures, predictors, potential confounders, and effect modifiers. Give diagnostic criteria, if applicable | Yes | Our Analysis section outlines how we calculated our two outcomes of interest: average nutrient content (subheading two) and adherence to targets (subheading three). |
| Data sources/ measurement | 8* | For each variable of interest, give sources of data and details of methods of assessment (measurement). Describe comparability of assessment methods if there is more than one group | Yes | The 'Data Collection' subheading of the Methods section (second subheading of the Methods) outlines the source of the nutritional data we collected. |
| Bias | 9 | Describe any efforts to address potential sources of bias | Yes | We acknowledge that bias may be resulting from our selection of the restaurants, as we are only focusing on the highest-grossing restaurant chains, and therefore our findings may not be representative of independent restaurants or smaller chains. In addition, the lack of sales weighting in our findings means that our assessment of a restaurant's target adherence may not be representative of the menu items that are actually being purchased by customers. |
| Study size | 10 | Explain how the study size was arrived at | Yes | The 'Identifying Restaurants' subheading of the Methods section (first subheading |

| | | | | |
|---|---|---|---|---|
| | | | | of Methods) outlines the criteria for the restaurants we included in our sample, and the first paragraph of the 'Missing Data' subheading in the Analysis section outlines our exclusion criteria for menu items. |
| Quantitative variables | 11 | Explain how quantitative variables were handled in the analyses. If applicable, describe which groupings were chosen and why | Yes | Under their respective subheadings in the Analysis section, we describe how our two outcome variables (average nutrient content under subheading two and target adherence under subheading three) were used for our analyses. |
| Statistical methods | 12 | (*a*) Describe all statistical methods, including those used to control for confounding | Yes | Our Analysis section outlines all analyses we conducted, however we did not include any inferential statistics in the paper, and we explain our reasoning in the ‘Deviations from Protocol’ section (subheading five of the Analysis section). |
| | | (*b*) Describe any methods used to examine subgroups and interactions | Yes | For some reporting of analyses, restaurants were assigned to one of five restaurant types. We describe the categorisation approach in the 'Categorising Menu Items' subheading of the Methods section (final subheading of Methods section). |
| | | (*c*) Explain how missing data were addressed | Yes | The 'Missing Data' subheading of the Analysis section explains how we dealt with missing data (first subheading of Analysis section). |
| | | (*d*) If applicable, describe analytical methods taking account of sampling strategy | | This study analysed nutritional data for all food items offered by the highest-grossing restaurant chains in the UK, and therefore was based on a ‘whole population’ of menu items, as supposed to a random sample. Considering this, |

| | | | | |
|---|---|---|---|---|
| | | | | we opted for a descriptive, rather than inferential analysis approach. |
| | | (*e*) Describe any sensitivity analyses | Yes | The sensitivity analyses we conducted is described under the 'Sensitivity Analyses' subheading of the Analysis section (subheading four of the Analysis section). |
| **Results** | | | | |
| Participants | 13* | (a) Report numbers of individuals at each stage of study—eg numbers potentially eligible, examined for eligibility, confirmed eligible, included in the study, completing follow-up, and analysed | Yes | In the first paragraph of the Results section, we provide the number of menu items excluded against each criterion, to go from our initially collected sample to our final sample of menu items. |
| | | (b) Give reasons for non-participation at each stage | | N/A |
| | | (c) Consider use of a flow diagram | Yes | Use of a flow diagram was not deemed appropriate. |
| Descriptive data | 14* | (a) Give characteristics of study participants (eg demographic, clinical, social) and information on exposures and potential confounders | Yes | Table 1 (following the second paragraph of the Results section) provides an overview of the characteristics for each restaurant, including Brand Name, Company Name, Restaurant Type, 2022 Sales Value, Number of Menu Items Included, and Number of Unique Subcategories Included. S2 Table also provides further details of the data collected for each restaurant. |
| | | (b) Indicate number of participants with missing data for each variable of interest | Yes | In the first paragraph of the Results section, we provide the number of menu items excluded against each criterion, to go from our initially collected sample to our final sample of menu items. |
| Outcome data | 15* | Report numbers of outcome events or summary measures | Yes | All outcomes by restaurant and subcategory are reported in supplementary tables. |

| | | | | |
|---|---|---|---|---|
| Main results | 16 | (*a*) Give unadjusted estimates and, if applicable, confounder-adjusted estimates and their precision (eg, 95% confidence interval). Make clear which confounders were adjusted for and why they were included | Yes | This is not relevant as we did not include any inferential statistics. |
| | | (*b*) Report category boundaries when continuous variables were categorized | | N/A |
| | | (*c*) If relevant, consider translating estimates of relative risk into absolute risk for a meaningful time period | | N/A |
| Other analyses | 17 | Report other analyses done—eg analyses of subgroups and interactions, and sensitivity analyses | Yes | Findings from the sensitivity analyses are reported from subheading three of the results section (titled 'Sensitivity Analysis Excluding Limited Time Offer Menu Items'), and full outcomes are reported in the supplementary material. |
| **Discussion** | | | | |
| Key results | 18 | Summarise key results with reference to study objectives | Yes | Key findings are summarised in the first paragraph of the Discussion. |
| Limitations | 19 | Discuss limitations of the study, taking into account sources of potential bias or imprecision. Discuss both direction and magnitude of any potential bias | Yes | Limitations of the study are described in the 'Strengths and Limitations' section of the discussion (subheading two of the Discussion). |
| Interpretation | 20 | Give a cautious overall interpretation of results considering objectives, limitations, multiplicity of analyses, results from similar studies, and other relevant evidence | Yes | We make considerations for how the limitations of the study may have impacted our findings in the 'Strengths and Limitations' section (subheading two of the Discussion), and interpret our results within the context of the wider literature in the 'Comparison to Other Literature' section (subheading one of the Discussion). |
| Generalisability | 21 | Discuss the generalisability (external validity) of the study results | Yes | We describe how our findings are generalisable beyond a UK context |

| | | | | |
|---|---|---|---|---|
| | | | | (paragraph three of the ‘Comparison to Other Literature’ subheading of the Discussion). |
| **Other information** | | | | |
| Funding | 22 | Give the source of funding and the role of the funders for the present study and, if applicable, for the original study on which the present article is based | Yes | Funding sources and role of funders in this study is provided on the title page. |
