# Supplementary material for: Adherence to voluntary UK sugar, salt, and calorie reduction targets in the highest-grossing restaurant chains: A cross-sectional study
Source: PLoS Med. 2026 May 5;23(5):e1004681. doi: 10.1371/journal.pmed.1004681 (PMC13143115; doi:10.1371/journal.pmed.1004681)
Supplement: S2 Table — Restaurants in descending order by number of menu items. (PDF) [file pmed.1004681.s003.pdf]

S2 Table – Overview of data collection approach and completeness of collected data, for each restaurant. Restaurants in descending order by number of menu items.

Restaurant	No. of Menu Items	Per 100g or Per Serving Information?	Serving Size (g) Provided?	Data Collection Method	Missing Nutrition Information	Notes
**Pizza Hut**	330	Majority per serving (per 100g only for other brand ice-creams)	Only for four products	Manual input from PDF	Kilojoules, Sodium, Fibre	
**Hungry Horse**	247	Per serving	No	PDF	Sodium, Fibre	
**Pizza Express**	247	Majority both (15 items with per 100g only)	No	PDF	Sodium	
**Domino's**	241	Majority both (per 100g only for other brand ice-creams)	No	PDF		
**Prezzo**	218	Per serving	No	PDF	Calories (had to calculate from fat, carbohydrates, and protein), Sodium	
**Toby Carvery**	214	Per serving	No	Web-scrape	Sodium, Fibre	When comparing kcal as reported to kcal as calculated from protein, fat, and carbohydrates, 17 products had a reported kcal that was at least 100kcal lower than the calculated kcal

						(ranging from a 114.68 to 936.98 difference), suggesting some inaccuracy in their reporting.
**Papa John's**	180	Majority both (37 items per 100g only)	Yes	Manual input from PDF		
**Harvester**	175	Per serving	For 13 items	PDF	Sodium, Fibre	
**Greggs**	174	Both	Yes	PDF	Sodium	
**Pret**	151	Majority both (12 items with per 100g only)	No	Web-scrape	Sodium	
**Costa**	123	Both	Yes	PDF	Sodium, some Fibre (only provided for other brand products)	
**McDonald's**	111	Per serving	No	Web-scrape	Sodium	
**Caffé Nero**	108	Both	Yes	PDF	Sodium, some Fibre	
**Wagamama**	105	Both	No	Web-scrape		
**Nando's**	90	Per serving	No	Web-scrape	Sodium	
**Subway**	80	Per serving	Yes	Web-scrape	Sodium	
**KFC**	76	Per serving	No	Web-scrape	Sodium, Fibre	
**Vintage Inns**	73	Per serving	No	PDF	Sodium, Fibre	
**Leon**	67	Per serving	Yes	Manual input from website	Kilojoules, Sodium	
**Starbucks**	49	Per serving	No	PDF	Sodium	
**Burger King**	40	Majority both (1 item per 100g only)	No	Manual input from website	Sodium, Fibre	In addition to their menu webpage, Burger King

						had a 'Nutrition Explorer' website. Nutritional information was taken from the menu webpage as this provided both per 100g and per serving information, whereas the Nutrition Explorer only provided per serving information.
