# Supplementary material for: Adherence to voluntary UK sugar, salt, and calorie reduction targets in the highest-grossing restaurant chains: A cross-sectional study
Source: PLoS Med. 2026 May 5;23(5):e1004681. doi: 10.1371/journal.pmed.1004681 (PMC13143115; doi:10.1371/journal.pmed.1004681)
Supplement: S3 Table — (PDF) [file pmed.1004681.s004.pdf]

S3 Table – Categorisation criteria for the 12 broad food subcategories.

Category	Subcategory	Definition
**Main meals**	Burgers	Burgers of all sizes
	Chicken	All main chicken dishes. Chicken products described as a 'side' to be included under 'other sides'.
	Pizzas	Pizzas of all sizes.
	Breakfast items	Any items described as breakfast items, such as bacon and egg sandwiches.
	Children's meals	Any main meal described as a kid’s meal. Main meals only, other products (e.g., sides) to be included in other categories accordingly.
	Sandwiches	All products described as a sandwich, wrap or slice, including hot sandwiches, paninis, pasties and sausage rolls.
	Salads	All salads as a main item. Side salad and green salads to be included under 'other sides'.
	Other mains	All other mains, including curries, fish and chips, roasts, and steaks.
**Sides/Extras**	Potato sides	All fries, chips, wedges and other potato side dishes
	Other sides	All other products described as a 'side', including side salads, soups and side portions of chicken etc
	Sauces	All sauces and condiments
**Desserts**	Desserts	Includes all desserts, cakes, pastries, ice creams and sweet treats
