# Supplementary material for: Adherence to voluntary UK sugar, salt, and calorie reduction targets in the highest-grossing restaurant chains: A cross-sectional study
Source: PLoS Med. 2026 May 5;23(5):e1004681. doi: 10.1371/journal.pmed.1004681 (PMC13143115; doi:10.1371/journal.pmed.1004681)
Supplement: S4 Table — (PDF) [file pmed.1004681.s005.pdf]

S4 Table – The range of calorie, salt (per 100g and per serving), and sugar target values set for menu items within each subcategory.

Subcategory	Calorie Target Values (per serving)	Salt Target Values (per 100g)	Salt Target Values (per serving)	Sugar Target Values (per 100g)
**Breakfast Items**	485	2.11	2.70	28.70
**Burgers**	380	0.00	2.20	
**Chicken**	0		3.15	
**Children's Meals**	485		0.09	0.00
**Desserts**		1.07		38.40
**Other Mains**	485	0.80	2.25	
**Other Sides**	745	2.16	3.15	0.00
**Pizzas**	0		5.12	
**Potato Sides**	260	0.53	0.47	
**Salads**	485			
**Sandwiches**	50		1.58	
**Sauces**		2.13		
