# Supplementary material for: Adherence to voluntary UK sugar, salt, and calorie reduction targets in the highest-grossing restaurant chains: A cross-sectional study
Source: PLoS Med. 2026 May 5;23(5):e1004681. doi: 10.1371/journal.pmed.1004681 (PMC13143115; doi:10.1371/journal.pmed.1004681)
Supplement: S5 Table — Subcategories are listed in descending order by the proportion of products belonging to that category where the subcategory mean serving size had to be used. (PDF) [file pmed.1004681.s006.pdf]

S5 Table – The number of products per subcategory where the subcategory mean serving size had to be used to calculate either per 100g or per serving nutrient information. Subcategories are listed in descending order by the proportion of products belonging to that category where the subcategory mean serving size had to be used.

Subcategory	No. of Products Affected	Proportion of Affected Products / Total Products (%)	Serving Size (g) for Products where Serving Size was Provided				
			Mean	SD	Median	Lower Quartile	Upper Quartile
**Chicken**	36	84	243.57	122.10	262.00	136.00	355.00
**Burgers**	62	72	239.39	62.96	242.48	217.02	262.27
**Sauces**	80	68	30.54	18.30	25.05	25.00	30.29
**Other Mains**	185	67	455.62	194.38	409.65	300.00	607.75
**Potato Sides**	73	63	111.83	67.23	111.00	40.00	155.47
**Other Sides**	363	62	114.29	68.87	109.00	60.89	158.30
**Breakfast Items**	133	59	167.48	69.33	164.50	110.75	208.88
**Children's Meals**	77	54	197.75	90.41	177.48	151.76	215.63
**Pizzas**	306	46	229.50	114.17	244.63	104.02	282.46
**Desserts**	232	45	78.14	49.48	74.53	48.26	100.00
**Salads**	18	39	219.54	95.65	192.00	147.25	295.83
**Sandwiches**	65	23	191.24	49.98	192.00	149.15	223.95
