# Supplementary material for: Adherence to voluntary UK sugar, salt, and calorie reduction targets in the highest-grossing restaurant chains: A cross-sectional study
Source: PLoS Med. 2026 May 5;23(5):e1004681. doi: 10.1371/journal.pmed.1004681 (PMC13143115; doi:10.1371/journal.pmed.1004681)
Supplement: S6 Table — Restaurants are listed in descending order by the proportion of products belonging to that category where the subcategory mean serving size had to be used. (PDF) [file pmed.1004681.s007.pdf]

S6 Table - The number of products per restaurant where the subcategory mean serving size had to be used to calculate either per 100g or per serving nutrient information. Restaurants are listed in descending order by the proportion of products belonging to that category where the subcategory mean serving size had to be used.

Restaurant	No. of Products Affected	Proportion of Affected Products / Total Products (%)	Serving Size (g) for Products where Serving Size was Provided				
			Mean	SD	Median	Lower Quartile	Upper Quartile
**Burger King**	1	3	179.30	85.53	150.87	108.17	246.89
**Caffé Nero**	0	0	104.00	74.22	93.50	45.00	133.25
**Costa**	0	0	90.86	63.87	78.00	41.50	130.00
**Domino's**	6	2	209.87	75.62	242.34	145.26	260.59
**Greggs**	0	0	142.18	74.02	149.35	76.85	200.78
**Harvester**	162	93	74.23	34.15	100.00	30.00	100.00
**Hungry Horse**	247	100	NA	NA	NA	NA	NA
**KFC**	76	100	NA	NA	NA	NA	NA
**Leon**	0	0	192.25	113.51	196.00	75.00	284.00
**McDonald's**	111	100	NA	NA	NA	NA	NA
**Nando's**	89	99	30.54	NA	30.54	30.54	30.54
**Papa John's**	37	21	113.15	62.55	93.88	84.15	112.92
**Pizza Express**	15	6	222.10	147.69	183.34	99.50	353.98
**Pizza Hut**	326	99	202.50	68.50	180.00	157.50	225.00
**Pret**	12	8	163.89	96.61	157.75	87.57	220.77
**Prezzo**	218	100	NA	NA	NA	NA	NA
**Starbucks**	49	100	NA	NA	NA	NA	NA
**Subway**	0	0	167.54	57.20	156.00	132.00	212.00
**Toby Carvery**	208	97	190.43	0.00	190.43	190.43	190.43
**Vintage Inns**	73	100	NA	NA	NA	NA	NA
**Wagamama**	0	0	364.63	248.99	294.63	141.77	603.33
