# Supplementary material for: Adherence to voluntary UK sugar, salt, and calorie reduction targets in the highest-grossing restaurant chains: A cross-sectional study
Source: PLoS Med. 2026 May 5;23(5):e1004681. doi: 10.1371/journal.pmed.1004681 (PMC13143115; doi:10.1371/journal.pmed.1004681)
Supplement: S7 Table — In descending order by Mean kcal per 100 g. (PDF) [file pmed.1004681.s008.pdf]

S7 Table - The mean, median, and standard deviation, for Kcal per 100g, per recommended serving, and per subcategory average serving, across all menu items in each subcategory. In descending order by Mean Kcal per 100g.

	Per 100g			Per Reported Serving			Per Subcategory Average Serving		
**Subcategory**	**Mean**	**SD**	**Median**	**Mean**	**SD**	**Median**	**Mean**	**SD**	**Median**
**Desserts**	409.48	275.79	390.00	314.18	248.04	280.00	319.96	215.50	304.74
**Potato Sides**	315.73	261.39	244.90	323.28	284.12	269.75	353.09	292.31	273.88
**Burgers**	308.36	193.85	256.60	737.24	471.38	603.60	738.18	464.06	614.27
**Sauces**	298.44	305.45	202.99	92.33	97.58	62.00	91.15	93.29	62.00
**Pizzas**	293.89	70.97	276.00	668.08	251.39	695.55	674.47	162.88	633.42
**Chicken**	280.77	197.96	212.87	680.66	487.79	568.00	683.87	482.17	518.50
**Sandwiches**	250.17	87.41	231.82	474.11	188.71	449.19	478.42	167.15	443.34
**Other Sides**	233.84	190.13	209.98	259.64	201.30	225.75	267.26	217.31	240.00
**Breakfast Items**	231.99	194.78	201.82	375.48	325.61	316.00	388.53	326.20	338.00
**Children's Meals**	196.37	96.53	175.49	371.59	188.22	343.50	388.33	190.89	347.03
**Other Mains**	167.35	82.37	157.81	755.83	387.52	738.00	762.50	375.28	719.00
**Salads**	145.91	79.66	134.50	334.34	210.58	310.00	320.33	174.88	295.28
