# Supplementary material for: Adherence to voluntary UK sugar, salt, and calorie reduction targets in the highest-grossing restaurant chains: A cross-sectional study
Source: PLoS Med. 2026 May 5;23(5):e1004681. doi: 10.1371/journal.pmed.1004681 (PMC13143115; doi:10.1371/journal.pmed.1004681)
Supplement: S10 Table — In descending order by Mean Fat per 100 g. (PDF) [file pmed.1004681.s011.pdf]

**S10 Table -** The mean, median, and standard deviation, for Fat per 100g, per recommended serving, and per subcategory average serving, across all menu items in each subcategory. In descending order by Mean Fat per 100g.

| | **Per 100g** | | | **Per Reported Serving** | | | **Per Subcategory Average Serving** | | |
|---|---|---|---|---|---|---|---|---|---|
| **Subcategory** | **Mean** | **SD** | **Median** | **Mean** | **SD** | **Median** | **Mean** | **SD** | **Median** |
| **Sauces** | 21.81 | 32.92 | 6.22 | 6.80 | 10.14 | 1.90 | 6.66 | 10.06 | 1.90 |
| **Desserts** | 19.58 | 16.31 | 17.40 | 14.92 | 13.29 | 12.99 | 15.30 | 12.74 | 13.60 |
| **Burgers** | 15.73 | 11.84 | 12.85 | 37.74 | 28.84 | 29.15 | 37.65 | 28.34 | 30.75 |
| **Potato Sides** | 14.09 | 10.40 | 10.77 | 13.96 | 10.12 | 12.00 | 15.76 | 11.63 | 12.04 |
| **Other Sides** | 12.84 | 14.22 | 10.50 | 14.21 | 15.61 | 10.65 | 14.68 | 16.25 | 12.00 |
| **Chicken** | 12.82 | 8.90 | 9.48 | 31.05 | 21.90 | 23.60 | 31.22 | 21.68 | 23.10 |
| **Pizzas** | 11.00 | 4.73 | 9.85 | 25.04 | 12.97 | 23.87 | 25.25 | 10.85 | 22.61 |
| **Sandwiches** | 10.60 | 6.51 | 9.10 | 20.02 | 12.60 | 17.94 | 20.27 | 12.46 | 17.40 |
| **Breakfast Items** | 9.85 | 10.87 | 7.90 | 16.51 | 18.26 | 12.00 | 16.50 | 18.20 | 13.23 |
| **Salads** | 8.27 | 5.50 | 7.45 | 19.30 | 14.11 | 17.30 | 18.17 | 12.08 | 16.36 |
| **Other Mains** | 7.96 | 4.94 | 6.78 | 35.47 | 22.04 | 32.05 | 36.27 | 22.53 | 30.90 |
| **Children's Meals** | 7.33 | 5.51 | 6.07 | 13.97 | 10.77 | 12.00 | 14.50 | 10.89 | 12.00 |
