# Supplementary material for: Adherence to voluntary UK sugar, salt, and calorie reduction targets in the highest-grossing restaurant chains: A cross-sectional study
Source: PLoS Med. 2026 May 5;23(5):e1004681. doi: 10.1371/journal.pmed.1004681 (PMC13143115; doi:10.1371/journal.pmed.1004681)
Supplement: S12 Table — In descending order by Mean Salt per 100 g. (PDF) [file pmed.1004681.s013.pdf]

S12 Table - The mean, median, and standard deviation, for Salt per 100g, per recommended serving, and per subcategory average serving, across all menu items in each restaurant. In descending order by Mean Salt per 100g.

	Per 100g			Per Reported Serving			Per Subcategory Average Serving		
**Restaurant**	**Mean**	**SD**	**Median**	**Mean**	**SD**	**Median**	**Mean**	**SD**	**Median**
**Prezzo**	1.81	1.55	1.36	3.67	2.69	3.60	3.67	2.69	3.60
**Papa John's**	1.61	2.77	1.38	1.78	3.25	1.29	2.83	3.19	2.85
**Domino's**	1.41	0.84	1.32	2.91	1.53	2.98	2.69	1.30	2.82
**Pizza Hut**	1.38	0.48	1.38	2.93	1.15	2.96	2.92	1.16	2.96
**Hungry Horse**	1.21	1.57	0.79	2.02	2.15	1.28	2.02	2.15	1.28
**Pizza Express**	1.16	1.05	1.10	2.63	2.29	2.08	1.93	1.56	1.94
**Nando's**	1.02	1.18	0.77	1.29	0.91	1.30	1.38	1.35	1.30
**Burger King**	1.02	0.43	1.10	1.92	1.23	1.84	2.02	1.12	2.11
**KFC**	0.98	1.12	0.72	1.38	1.25	0.92	1.38	1.25	0.92
**Greggs**	0.95	0.63	0.83	1.24	0.80	1.30	1.47	0.99	1.47
**Subway**	0.95	0.37	0.95	1.58	0.79	1.50	1.70	0.70	1.75
**Harvester**	0.93	0.95	0.72	1.72	2.09	1.05	1.76	2.08	1.06
**Vintage Inns**	0.90	0.80	0.68	1.85	1.37	1.63	1.85	1.37	1.63
**Pret**	0.82	0.59	0.78	1.39	1.07	1.41	1.57	1.21	1.53
**Toby Carvery**	0.81	1.38	0.39	1.02	1.47	0.51	1.09	1.77	0.51
**Leon**	0.78	0.50	0.72	1.38	1.05	1.30	1.39	1.17	1.28
**Wagamama**	0.75	0.65	0.62	2.54	2.18	2.28	1.95	1.48	1.73
**McDonald's**	0.73	0.52	0.58	1.21	0.94	1.10	1.21	0.94	1.10
**Costa**	0.62	0.43	0.55	0.63	0.63	0.38	0.77	0.72	0.45
**Caffé Nero**	0.61	0.46	0.52	0.71	0.75	0.46	0.86	0.89	0.46
**Starbucks**	0.54	0.39	0.47	0.67	0.67	0.40	0.67	0.67	0.40
