# Supplementary material for: Adherence to voluntary UK sugar, salt, and calorie reduction targets in the highest-grossing restaurant chains: A cross-sectional study
Source: PLoS Med. 2026 May 5;23(5):e1004681. doi: 10.1371/journal.pmed.1004681 (PMC13143115; doi:10.1371/journal.pmed.1004681)
Supplement: S13 Table — In descending order by Mean Sugar per 100 g. (PDF) [file pmed.1004681.s014.pdf]

S13 Table - The mean, median, and standard deviation, for Sugar per 100g, per recommended serving, and per subcategory average serving, across all menu items in each restaurant. In descending order by Mean Sugar per 100g.

	Per 100g			Per Reported Serving			Per Subcategory Average Serving		
**Restaurant**	**Mean**	**SD**	**Median**	**Mean**	**SD**	**Median**	**Mean**	**SD**	**Median**
**Costa**	21.99	17.48	23.10	13.52	12.29	7.64	18.34	13.08	18.75
**Caffé Nero**	20.68	17.45	20.80	14.63	11.35	11.88	17.80	12.80	18.03
**Vintage Inns**	15.74	29.17	3.09	16.33	22.17	5.80	16.33	22.17	5.80
**Harvester**	15.34	26.13	5.86	17.23	22.29	8.30	18.58	23.54	9.10
**Starbucks**	15.24	13.87	12.03	13.37	10.04	12.00	13.37	10.04	12.00
**Toby Carvery**	12.49	19.40	3.85	11.45	15.15	4.85	11.64	15.17	5.00
**Hungry Horse**	10.54	29.22	3.27	12.50	23.51	5.90	12.50	23.51	5.90
**Greggs**	10.40	13.01	3.80	10.66	13.47	6.64	10.28	9.13	7.27
**Pret**	9.52	12.57	3.40	9.86	9.05	6.22	10.88	10.24	7.09
**Nando's**	9.44	16.35	2.14	8.60	12.41	4.20	8.76	12.46	4.20
**McDonald's**	9.32	12.81	3.66	10.08	9.78	7.30	10.08	9.78	7.30
**KFC**	9.01	13.24	4.23	8.88	9.67	6.45	8.88	9.67	6.45
**Leon**	7.21	9.66	3.09	8.16	7.03	6.00	7.93	6.97	5.87
**Prezzo**	6.99	13.16	2.85	10.05	10.83	7.10	10.05	10.83	7.10
**Domino's**	6.65	7.06	6.00	12.41	7.75	14.16	11.61	7.13	13.54
**Pizza Express**	6.51	10.21	3.10	10.00	11.46	5.77	7.82	8.48	5.05
**Papa John's**	6.09	6.21	5.00	7.19	14.02	4.55	10.07	5.67	11.25
**Burger King**	5.30	6.45	3.70	8.01	6.13	7.09	8.20	5.42	7.78
**Wagamama**	5.19	5.70	3.09	12.52	9.24	10.56	9.54	5.95	7.97
**Pizza Hut**	4.27	5.50	2.96	7.50	4.64	6.60	7.50	4.65	6.60
**Subway**	3.37	1.62	3.59	5.93	4.21	5.10	6.23	3.17	6.68
