# Supplementary material for: Adherence to voluntary UK sugar, salt, and calorie reduction targets in the highest-grossing restaurant chains: A cross-sectional study
Source: PLoS Med. 2026 May 5;23(5):e1004681. doi: 10.1371/journal.pmed.1004681 (PMC13143115; doi:10.1371/journal.pmed.1004681)
Supplement: S14 Table — In descending order by Mean Fat per 100 g. (PDF) [file pmed.1004681.s015.pdf]

S14 Table - The mean, median, and standard deviation, for Fat per 100g, per recommended serving, and per subcategory average serving, across all menu items in each restaurant. In descending order by Mean Fat per 100g.

	Per 100g			Per Reported Serving			Per Subcategory Average Serving		
**Restaurant**	**Mean**	**SD**	**Median**	**Mean**	**SD**	**Median**	**Mean**	**SD**	**Median**
**Vintage Inns**	19.30	20.15	12.07	29.91	20.31	24.90	29.91	20.31	24.90
**Prezzo**	18.75	23.10	13.28	32.58	24.56	29.00	32.58	24.56	29.00
**Caffé Nero**	18.71	12.69	17.95	14.80	7.45	14.63	19.65	12.59	17.88
**Costa**	15.49	9.60	15.00	12.53	8.34	13.05	15.50	9.07	14.92
**Harvester**	15.06	14.88	11.58	26.19	25.94	16.70	26.40	25.81	17.00
**Hungry Horse**	14.46	17.35	9.31	25.40	26.65	16.20	25.40	26.65	16.20
**Nando's**	14.05	16.29	10.08	16.70	12.30	15.35	17.69	16.49	15.35
**Starbucks**	13.95	8.97	11.37	13.51	6.34	14.00	13.51	6.34	14.00
**Toby Carvery**	13.41	19.38	6.31	16.42	22.45	8.00	17.19	23.71	8.00
**Pret**	13.05	9.39	11.20	16.71	9.54	14.97	19.31	9.96	18.67
**Burger King**	12.93	2.80	12.70	24.06	14.36	20.78	24.60	10.89	26.57
**Pizza Hut**	11.83	4.28	10.89	24.46	9.88	22.33	24.40	9.97	22.33
**Leon**	11.70	8.64	9.43	17.39	9.52	16.00	17.14	11.05	17.24
**Greggs**	11.62	8.29	9.55	14.03	9.67	12.58	16.56	12.86	14.73
**Domino's**	11.01	7.37	10.50	20.98	8.89	19.22	19.59	7.38	18.59
**Papa John's**	10.83	6.40	9.25	12.51	11.61	8.81	18.69	8.61	17.56
**Pizza Express**	10.70	11.14	7.40	18.63	14.00	15.09	14.06	9.25	13.77
**KFC**	9.09	7.98	7.89	12.41	9.65	10.95	12.41	9.65	10.95
**McDonald's**	7.83	6.81	6.57	12.41	9.99	11.30	12.41	9.99	11.30
**Subway**	7.68	4.57	7.63	13.02	8.71	13.00	13.13	7.25	13.39
**Wagamama**	7.39	5.79	6.14	22.25	15.64	19.06	17.15	11.68	15.26
