# Supplementary material for: Adherence to voluntary UK sugar, salt, and calorie reduction targets in the highest-grossing restaurant chains: A cross-sectional study
Source: PLoS Med. 2026 May 5;23(5):e1004681. doi: 10.1371/journal.pmed.1004681 (PMC13143115; doi:10.1371/journal.pmed.1004681)
Supplement: S17 Table — In descending order by Mean Sugar per 100 g. (PDF) [file pmed.1004681.s018.pdf]

S17 Table - The mean, median, and standard deviation, for Sugar per 100g, per recommended serving, and per subcategory average serving, across all menu items in each restaurant group. In descending order by Mean Sugar per 100g.

	**Per 100g**			**Per Reported Serving**			**Per Subcategory Average Serving**		
**Restaurant Type**	**Mean**	**SD**	**Median**	**Mean**	**SD**	**Median**	**Mean**	**SD**	**Median**
**Sandwich**	13.43	15.25	4.50	11.27	11.27	6.95	12.79	11.20	8.25
**Other Main**	10.61	21.99	3.22	12.56	17.91	6.90	12.51	18.12	6.80
**Chicken**	9.24	14.96	2.99	8.73	11.21	5.00	8.81	11.24	5.00
**Burger**	8.26	11.59	3.66	9.53	8.99	7.30	9.58	8.86	7.42
**Pizza**	5.73	7.46	3.63	9.25	9.68	6.50	9.03	6.77	7.66
