# Supplementary material for: Adherence to voluntary UK sugar, salt, and calorie reduction targets in the highest-grossing restaurant chains: A cross-sectional study
Source: PLoS Med. 2026 May 5;23(5):e1004681. doi: 10.1371/journal.pmed.1004681 (PMC13143115; doi:10.1371/journal.pmed.1004681)
Supplement: S19 Table — In descending order by proportion of menu items meeting all applicable targets. (PDF) [file pmed.1004681.s020.pdf]

S19 Table - The proportion of menu items meeting sugar, salt, calorie, and all applicable targets, for each restaurant group. In descending order by proportion of menu items meeting all applicable targets.

	Calorie Targets			Salt Targets			Sugar Targets			All Applicable Targets		
**Restaurant Type**	**No. of eligible menu items**	**Proportion of menu items meeting target (%)**	**Range in proportion across restaurants (%)**	**No. of eligible menu items**	**Proportion of menu items meeting target (%)**	**Range in proportion across restaurants (%)**	**No. of eligible menu items**	**Proportion of menu items meeting target (%)**	**Range in proportion across restaurants (%)**	**No. of eligible menu items**	**Proportion of menu items meeting target (%)**	**Range in proportion across restaurants (%)**
**Burger**	102	69	42	115	80	10	36	53	59	149	59	33
**Chicken**	124	78	20	116	66	6	23	0	0	166	58	5
**Sandwich**	369	72	20	566	69	33	257	35	60	657	48	47
**Other Main**	707	59	42	715	53	53	197	42	63	1018	45	37
**Pizza**	846	53	27	832	49	60	65	23	19	961	32	33
