# Supplementary material for: Adherence to voluntary UK sugar, salt, and calorie reduction targets in the highest-grossing restaurant chains: A cross-sectional study
Source: PLoS Med. 2026 May 5;23(5):e1004681. doi: 10.1371/journal.pmed.1004681 (PMC13143115; doi:10.1371/journal.pmed.1004681)
Supplement: S20 Table — (PDF) [file pmed.1004681.s021.pdf]

S20 Table - Mean nutrient content per 100g and per serving for restaurants with limited time menu items, including (as per primary analysis) and excluding the limited time offer items.

Restaurant	Kcal per 100g		Salt per 100g		Sugar per 100g		Kcal per Serving		Salt per Serving		Sugar per Serving	
	Inc.	Exc.	Inc.	Exc.	Inc.	Exc.	Inc.	Exc.	Inc.	Exc.	Inc.	Exc.
**McDonald's**	194.57	185.19	0.73	0.72	9.32	8.63	308.46	298.01	1.21	1.18	10.08	9.58
**Burger King**	252.75	251.56	1.02	1.00	5.30	5.30	459.85	446.41	1.92	1.84	8.01	7.71
**Pret**	262.78	269.03	0.82	0.82	9.52	9.97	344.69	337.59	1.39	1.36	9.86	9.87
**KFC**	206.14	218.37	0.98	1.04	9.01	10.78	291.05	288.36	1.38	1.35	8.88	10.02
