# Supplementary material for: Adherence to voluntary UK sugar, salt, and calorie reduction targets in the highest-grossing restaurant chains: A cross-sectional study
Source: PLoS Med. 2026 May 5;23(5):e1004681. doi: 10.1371/journal.pmed.1004681 (PMC13143115; doi:10.1371/journal.pmed.1004681)
Supplement: S21 Table — (PDF) [file pmed.1004681.s022.pdf]

S21 Table - The proportion of menu items meeting sugar, salt, calorie, and all applicable targets, for restaurants with limited time menu items, including and excluding limited time offer items.

Restaurant	Calorie Targets (%)		Salt Targets (%)		Sugar Targets (%)		All Applicable (%)	
	Inc.	Exc.	Inc.	Exc.	Inc.	Exc.	Inc.	Exc.
**McDonald's**	83	84	83	83	59	66	68	69
**Burger King**	42	44	73	74	0	0	35	37
**Pret**	70	69	67	65	34	32	53	51
**KFC**	89	85	63	52	0	0	61	52
