# Supplementary material for: Adherence to voluntary UK sugar, salt, and calorie reduction targets in the highest-grossing restaurant chains: A cross-sectional study
Source: PLoS Med. 2026 May 5;23(5):e1004681. doi: 10.1371/journal.pmed.1004681 (PMC13143115; doi:10.1371/journal.pmed.1004681)
Supplement: S22 Table — Subcategories are listed in descending order by mean kcal per 100 g. (PDF) [file pmed.1004681.s023.pdf]

S22 Table – Mean nutrient content per 100g for each subcategory when the subcategory average (as per the primary analysis), lower quartile, and upper quartile, were used to replace missing serving size. Subcategories are listed in descending order by mean kcal per 100g.

Subcategory	Kcal per 100g			Salt per 100g			Sugar per 100g		
	Mean	Upper Q	Lower Q	Mean	Upper Q	Lower Q	Mean	Upper Q	Lower Q
**Desserts**	409.48	368.90	524.41	0.48	0.43	0.61	34.24	30.51	44.78
**Potato Sides**	315.73	259.21	677.34	0.76	0.65	1.45	2.07	1.61	5.01
**Burgers**	308.36	287.98	332.45	1.18	1.11	1.27	4.25	3.98	4.58
**Sauces**	298.44	300.18	344.00	2.19	2.21	2.54	15.29	15.38	17.65
**Pizzas**	293.89	265.11	479.00	1.50	1.36	2.40	4.52	4.21	6.50
**Chicken**	280.77	203.17	476.31	1.08	0.78	1.84	3.96	2.78	6.95
**Sandwiches**	250.17	240.50	268.85	1.11	1.07	1.20	3.65	3.51	3.92
**Other Sides**	233.84	197.91	347.18	1.33	1.15	1.91	3.96	3.35	5.90
**Breakfast Items**	231.99	204.67	302.61	0.99	0.86	1.31	6.75	6.02	8.64
**Children's Meals**	196.37	187.30	229.55	0.88	0.84	1.03	3.17	3.01	3.78
**Other Mains**	167.35	136.98	230.30	0.76	0.63	1.02	2.63	2.19	3.56
**Salads**	145.91	128.33	179.38	0.75	0.67	0.90	2.62	2.34	3.15
