# Supplementary material for: Adherence to voluntary UK sugar, salt, and calorie reduction targets in the highest-grossing restaurant chains: A cross-sectional study
Source: PLoS Med. 2026 May 5;23(5):e1004681. doi: 10.1371/journal.pmed.1004681 (PMC13143115; doi:10.1371/journal.pmed.1004681)
Supplement: S23 Table — Subcategories are listed in descending order by mean kcal per serving. (PDF) [file pmed.1004681.s024.pdf]

S23 Table – Mean nutrient content per serving for each subcategory when the subcategory average (as per the primary analysis), lower quartile, and upper quartile, were used to replace missing serving size. Subcategories are listed in descending order by mean kcal per serving.

Subcategory	Kcal per Serving			Salt per Serving			Sugar per Serving		
	**Mean**	**Upper Q**	**Lower Q**	**Mean**	**Upper Q**	**Lower Q**	**Mean**	**Upper Q**	**Lower Q**
**Other Mains**	755.83	757.25	754.37	3.44	3.44	3.43	12.05	12.09	12.00
**Burgers**	737.24	737.92	736.57	2.83	2.83	2.82	10.15	10.16	10.13
**Chicken**	680.66	680.66	680.66	2.63	2.63	2.63	9.71	9.71	9.71
**Pizzas**	668.08	668.08	668.08	3.44	3.44	3.44	10.11	10.11	10.11
**Sandwiches**	474.11	475.98	471.71	2.12	2.13	2.11	7.14	7.17	7.10
**Breakfast Items**	375.48	375.66	375.25	1.64	1.64	1.64	10.32	10.34	10.30
**Children's Meals**	371.59	371.59	371.59	1.66	1.66	1.66	6.10	6.10	6.10
**Salads**	334.34	339.81	329.15	1.63	1.65	1.62	6.01	6.10	5.92
**Potato Sides**	323.28	323.28	323.28	0.81	0.81	0.81	2.21	2.21	2.21
**Desserts**	314.18	316.75	310.66	0.39	0.39	0.39	26.18	26.41	25.88
**Other Sides**	259.64	266.18	251.69	1.48	1.57	1.37	4.34	4.42	4.24
**Sauces**	92.33	92.32	92.22	0.66	0.66	0.66	4.46	4.46	4.45
