# Supplementary material for: Adherence to voluntary UK sugar, salt, and calorie reduction targets in the highest-grossing restaurant chains: A cross-sectional study
Source: PLoS Med. 2026 May 5;23(5):e1004681. doi: 10.1371/journal.pmed.1004681 (PMC13143115; doi:10.1371/journal.pmed.1004681)
Supplement: S24 Table — Restaurants are listed in descending order by mean kcal per 100 g. (PDF) [file pmed.1004681.s025.pdf]

S24 Table – Mean nutrient content per 100g for each restaurant when the subcategory average (as per the primary analysis), lower quartile, and upper quartile, were used to replace missing serving size. Restaurants are listed in descending order by mean kcal per 100g.

Restaurant	Kcal per 100g			Salt per 100g			Sugar per 100g		
	Mean	Upper Q	Lower Q	Mean	Upper Q	Lower Q	Mean	Upper Q	Lower Q
**Caffé Nero**	367.85	367.85	367.85	0.61	0.61	0.61	20.68	20.68	20.68
**Vintage Inns**	358.38	280.64	620.56	0.90	0.71	1.56	15.74	12.33	26.05
**Costa**	355.23	355.23	355.23	0.62	0.62	0.62	21.99	21.99	21.99
**Prezzo**	332.14	268.80	572.98	1.81	1.43	3.27	6.99	5.56	11.78
**Harvester**	318.55	250.05	527.21	0.93	0.73	1.54	15.34	12.27	24.20
**Hungry Horse**	312.19	248.03	513.28	1.21	1.01	1.83	10.54	8.61	16.31
**Pizza Hut**	302.70	245.71	638.82	1.38	1.12	2.92	4.27	3.52	8.22
**Starbucks**	300.88	236.39	493.97	0.54	0.43	0.87	15.24	11.94	24.54
**Nando's**	291.18	237.14	464.08	1.02	0.84	1.60	9.44	7.73	14.69
**Greggs**	271.75	271.75	271.75	0.95	0.95	0.95	10.40	10.40	10.40
**Domino's**	265.75	265.75	265.75	1.41	1.41	1.41	6.65	6.65	6.65
**Pret**	262.78	262.78	262.78	0.82	0.82	0.82	9.52	9.52	9.52
**Papa John's**	260.19	260.19	260.19	1.61	1.61	1.61	6.09	6.09	6.09
**Burger King**	252.75	252.75	252.75	1.02	1.02	1.02	5.30	5.30	5.30
**Toby Carvery**	248.52	195.59	428.91	0.81	0.67	1.30	12.49	9.95	20.28
**Pizza Express**	224.13	224.13	224.13	1.16	1.16	1.16	6.51	6.51	6.51
**Leon**	223.03	223.03	223.03	0.78	0.78	0.78	7.21	7.21	7.21
**KFC**	206.14	169.85	328.05	0.98	0.84	1.46	9.01	7.47	13.78
**Subway**	196.55	196.55	196.55	0.95	0.95	0.95	3.37	3.37	3.37
**McDonald's**	194.57	162.00	284.99	0.73	0.63	1.01	9.32	7.91	13.66
**Wagamama**	161.78	161.78	161.78	0.75	0.75	0.75	5.19	5.19	5.19
