# Supplementary material for: Adherence to voluntary UK sugar, salt, and calorie reduction targets in the highest-grossing restaurant chains: A cross-sectional study
Source: PLoS Med. 2026 May 5;23(5):e1004681. doi: 10.1371/journal.pmed.1004681 (PMC13143115; doi:10.1371/journal.pmed.1004681)
Supplement: S26 Table — Subcategories are listed in descending order by proportion of menu items meeting all applicable targets when the average serving size was used to replace missing values. (PDF) [file pmed.1004681.s027.pdf]

S26 Table - The proportion of menu items meeting sugar, salt, calorie, and all applicable targets for each subcategory, when the subcategory average (as per the primary analysis), lower quartile, and upper quartile, were used to replace missing serving size. Subcategories are listed in descending order by proportion of menu items meeting all applicable targets when the average serving size was used to replace missing values.

Subcategory	Proportion of Menu Items Meeting Calorie Targets (%)			Proportion of Menu Items Meeting Salt Targets (%)			Proportion of Menu Items Meeting Sugar Targets (%)			Proportion of Menu Items Meeting All Applicable Targets (%)		
	Avg.	Up. Q	Low Q	Avg.	Up. Q	Low Q	Avg.	Up. Q	Low Q	Avg.	Up. Q	Low Q
**Salads**	96	96	96	NA	NA	NA	NA	NA	NA	96	96	96
**Breakfast Items**	61	61	61	82	82	82	74	76	71	66	67	64
**Chicken**	72	72	72	81	81	81	NA	NA	NA	65	65	65
**Other Sides**	75	75	75	64	64	64	100	100	100	63	65	61
**Children's Meals**	95	95	95	62	62	62	100	100	100	61	61	61
**Sandwiches**	59	59	60	76	76	76	NA	NA	NA	56	55	57
**Potato Sides**	59	59	59	71	71	71	NA	NA	NA	44	44	44
**Burgers**	52	52	52	73	73	73	NA	NA	NA	42	42	42
**Sauces**	NA	NA	NA	42	42	42	NA	NA	NA	42	42	37
**Other Mains**	62	62	62	45	45	45	NA	NA	NA	39	42	39
**Pizzas**	43	43	43	50	50	50	NA	NA	NA	25	25	25
**Desserts**	NA	NA	NA	40	40	40	30	34	24	22	25	17
