# Supplementary material for: Adherence to voluntary UK sugar, salt, and calorie reduction targets in the highest-grossing restaurant chains: A cross-sectional study
Source: PLoS Med. 2026 May 5;23(5):e1004681. doi: 10.1371/journal.pmed.1004681 (PMC13143115; doi:10.1371/journal.pmed.1004681)
Supplement: S27 Table — Restaurants are listed in descending order by proportion of menu items meeting all applicable targets when the average serving size was used to replace missing values. (PDF) [file pmed.1004681.s028.pdf]

S27 Table - The proportion of menu items meeting sugar, salt, calorie, and all applicable targets for each restaurant, when the subcategory average (as per the primary analysis), lower quartile, and upper quartile, were used to replace missing serving size. Restaurants are listed in descending order by proportion of menu items meeting all applicable targets when the average serving size was used to replace missing values.

Restaurant	Proportion of Menu Items Meeting Calorie Targets (%)			Proportion of Menu Items Meeting Salt Targets (%)			Proportion of Menu Items Meeting Sugar Targets (%)			Proportion of Menu Items Meeting All Applicable Targets (%)		
	Avg.	Up. Q	Low Q	Avg.	Up. Q	Low Q	Avg.	Up. Q	Low Q	Avg.	Up. Q	Low Q
**Subway**	80	80	80	89	89	89	0	0	0	76	76	76
**McDonald's**	83	83	83	83	84	78	59	78	47	68	72	64
**Toby Carvery**	73	73	73	72	76	66	47	51	44	63	65	59
**Leon**	86	86	86	65	65	65	63	63	63	63	63	63
**KFC**	89	89	89	63	65	61	0	11	0	61	63	61
**Nando's**	70	70	70	69	71	61	0	21	0	56	59	51
**Pret**	70	67	72	67	66	68	34	34	34	53	52	55
**Greggs**	75	75	75	73	73	73	46	46	46	52	52	52
**Starbucks**	60	60	60	66	76	51	60	80	30	51	60	38
**Wagamama**	77	77	77	53	53	53	46	46	46	49	49	49
**Harvester**	60	60	60	63	66	61	32	34	27	47	48	45
**Hungry Horse**	49	49	49	57	60	54	45	45	27	42	45	39
**Pizza Hut**	54	54	54	68	68	68	33	33	25	41	41	41
**Vintage Inns**	59	59	59	58	68	53	0	0	0	40	47	36
**Burger King**	42	42	42	73	73	76	0	0	0	35	35	35
**Domino's**	61	61	61	53	53	53	27	27	27	35	35	35
**Pizza Express**	55	55	55	45	45	45	14	14	14	35	35	35
**Costa**	69	69	69	66	66	66	26	26	26	31	31	31
**Caffé Nero**	63	63	63	56	56	56	27	27	27	30	30	30
**Prezzo**	44	44	44	19	21	17	48	61	13	26	29	21
**Papa John's**	35	35	35	8	8	8	29	29	29	8	8	8
