# Supplementary material for: Adherence to voluntary UK sugar, salt, and calorie reduction targets in the highest-grossing restaurant chains: A cross-sectional study
Source: PLoS Med. 2026 May 5;23(5):e1004681. doi: 10.1371/journal.pmed.1004681 (PMC13143115; doi:10.1371/journal.pmed.1004681)
Supplement: Attachment — Submitted filename: Reviewer responses.docx [file pmed.1004681.s032.docx]

Requests from Editors:

GENERAL EDITORIAL REQUESTS

* Please confirm that your title complies with PLOS Medicine's style. Your title must be nondeclarative and not a question. It should begin with main concept if possible. "Effect of" should be used only if causality can be inferred, i.e., for an RCT. Please place the study design ("A randomized controlled trial," "A retrospective study," "A modelling study," etc.) in the subtitle (ie, after a colon).

Confirmed

* Please confirm that your abstract complies with our requirements, including format (three sections: Background, Methods and Findings, and Conclusions) and providing all the information relevant to this study type <https://journals.plos.org/plosmedicine/s/submission-guidelines#loc-abstract>

We have added the key limitation of the study to the abstract ‘methods/findings’ section (line 67).

* Please ensure that the Introduction ends with a clear description of the study question or hypothesis.

Confirmed

* Please ensure that all abbreviations are defined at first use throughout the text.

Confirmed

* Please confirm that all numbers presented in the abstract are present and identical to numbers presented in the main manuscript text.

Confirmed

GENERAL

* Please review your text for claims of novelty or primacy (e.g. 'for the first time') and remove this language. In addition, please check that any use of statistical terms (such as trend or significant) are supported by the data, and if not please remove them.

We have corrected the use of novelty/’first’ language by changing the following sentence from “This study is the first to evaluate all three reduction programmes in a single study” to “We evaluated all three reduction programmes in a single study”. The second comment does not apply as we did not include any statistical testing (line 605).

* Statistical reporting: Please revise throughout the manuscript, including tables and figures.

Not applicable as we did not include any statistical testing.

- Please report statistical information as follows to improve clarity for the reader ""22% (95% CI [13,28]; p</=)"".

Not applicable as we did not include any statistical testing.

- Please separate upper and lower bounds with commas instead of hyphens as the latter can be confused with reporting of negative values.

Not applicable as we did not include any statistical testing.

- Please repeat statistical definitions (HR, CI etc.) for each set of parentheses."

Not applicable as we did not include any statistical testing.

* In the last sentence of the Abstract Methods and Findings section, please describe the main limitation(s) of the study's methodology.

We have added the key limitation of the study to the last sentence of the abstract’s ‘methods/findings’ section (line 67).

* In the abstract, please include the important dependent variables that are adjusted for in the analyses.

Not applicable as we did not include any statistical testing.

FUNDING STATEMENT

* The funding statement should include: specific grant numbers, initials of authors who received each award, URLs to sponsors’ websites. Also, please state whether any sponsors or funders (other than the named authors) played any role in study design, data collection and analysis, the decision to publish, or preparation of the manuscript. If they had no role in the research, include this sentence: “The funders had no role in study design, data collection and analysis, decision to publish, or preparation of the manuscript.”

We have added URLs to sponsors’ websites. There is no grant number associated with the NIHR Oxford Health Biomedical Research Centre or the NIHR Applied Research Collaboration (ARC) Oxford and Thames Valley.

* It appears that one or more study authors is affiliated with one or more of the agencies that funded the study. Thus, the statement “The funders had no role in study design, data collection and analysis, decision to publish, or preparation of the manuscript” does not apply. Please revise the Financial Disclosure accordingly, as in "[Author name] is [author's role] at [funding agency]. The funders had no other role in study design…..”

We believe this is a mistake as all authors are only affiliated with the University of Oxford, which is not one of the listed funders. Therefore, we have kept the financial disclosure the same.

COMPETING INTERESTS STATEMENT

* All authors must declare their relevant competing interests per the PLOS policy, which can be seen here: <https://journals.plos.org/plosmedicine/s/competing-interests> For authors with ties to industry, please indicate whether any of the interests has a financial stake in the results of the current study.

Confirmed

DATA AVAILABILITY

* The Data Availability Statement (DAS) requires revision. For each data source used in your study:

a) If the data are freely or publicly available, note this and state the location of the data: within the paper, in Supporting Information files, or in a public repository (include the DOI or accession number).

We have now included the phrase ‘publicly available’ (line 33).

b) If the data are owned by a third party but freely available upon request, please note this and state the owner of the data set and contact information for data requests (web or email address). Note that a study author cannot be the contact person for the data.

NA

c) If the data are not freely available, please describe briefly the ethical, legal, or contractual restriction that prevents you from sharing it. Please also include an appropriate contact (web or email address) for inquiries (again, this cannot be a study author).

NA

ETHICS AND CONSENT

* Please provide the name(s) of the institutional review board(s) that provided ethical approval. If ethics approval and/or informed consent were not required please explain this stating the reason why in the Methods section.

We have added the following sentence about ethical approval not being needed: “Ethical approval was not required for this study as it does not involve human participants.” (line 192).

FIGURES

* Please define all elements of box plots in the figure caption - center line, box limits and whiskers.

The definition of elements of box plots has been moved from figure titles to figure captions (for figures 2, 3, 4, and 5).

* Please provide titles and legends for all figures and tables (including those in Supporting Information files). Please define all acronyms used in each figure or table in its corresponding legend.

Confirmed

* Please ensure that where relevant figures include 95% CIs.

Not applicable as we did not include any statistical testing.

OBSERVATIONAL, COHORT, CROSS-SECTIONAL, AND CASE CONTROL STUDIES

* Please ensure that the study is reported according to the STROBE guideline, and include the completed STROBE checklist as Supporting Information. Please add the following statement, or similar, to the Methods: ""This study is reported as per the Strengthening the Reporting of Observational Studies in Epidemiology (STROBE) guideline (S1 Checklist).""

The STROBE guideline can be found here: <http://www.equator-network.org/reporting-guidelines/strobe/>

Confirmed

When completing the checklist, please use section and paragraph numbers, rather than page numbers.

Confirmed

* Did your study have a prospective protocol or analysis plan? Please state this (either way) early in the Methods section.

Confirmed

a) If a prospective analysis plan (from your funding proposal, IRB or other ethics committee submission, study protocol, or other planning document written before analyzing the data) was used in designing the study, please include the relevant prospectively written document with your revised manuscript as a Supporting Information file to be published alongside your study, and cite it in the Methods section. A legend for this file should be included at the end of your manuscript.

The study protocol has been added as S1 File and is referenced in the manuscript, and has been added to the supplementary file title list at the end of the manuscript.

b) If no such document exists, please make sure that the Methods section transparently describes when analyses were planned, and when/why any data-driven changes to analyses took place.

NA

b) In either case, changes in the analysis-- including those made in response to peer review comments-- should be identified as such in the Methods section of the paper, with rationale.

Confirmed

* Your study is observational and therefore causality cannot be inferred. Please remove language that implies causality and refer to associations instead.

Confirmed

* For all observational studies, in the manuscript text, please indicate: (1) the specific hypotheses you intended to test, (2) the analytical methods by which you planned to test them, (3) the analyses you actually performed, and (4) when reported analyses differ from those that were planned, transparent explanations for differences that affect the reliability of the study's results. If a reported analysis was performed based on an interesting but unanticipated pattern in the data, please be clear that the analysis was data-driven.

Confirmed

Comments from Reviewers:

Reviewer #1: The authors have fairly comprehensively addressed my comments to the original manuscript. I have no major comments remaining.

MINOR COMMENTS / TYPOS

l. 320: This is at the discretion of the editor (if there is a journal policy regarding singular or plural use of 'data') and/or the authors (if there is not): while 'data' can be found in the literature being used both as a singular and a plural, I do think the plural is more common and more correct (both in the sense that there is more than one data point in most situations and also given that 'data' is the plural of the latin 'datum') - as such, I would recommend ammending this sentence to "the data are not a random sample" or alternatively "the dataset is not a random sample".

Thank you for the suggestion, ‘data’ has been changed to ‘dataset’ (line 331).

l. 321-324: Not sure this really needs stating so explicitly in the final version of the manuscript, but if you do keep it in, then I would suggest changing the word 'true' to 'real' as I think it is slightly more appropriate. I apologise if the choice of 'true' is due to my choice of words in my original review. Even simpler, and shorter, perhaps, you could simply state that the observed differences are not due to sampling variation.

Thank you, we think that it is important to keep in this explanation as it is a major deviation from the planned analysis, so we have followed your suggestion of changing ‘true’ to ‘real’ (line 333).

Reviewer #2: The authors were responsive to my comments and have improved the manuscript. One minor remaining item that the authors should clarify is precisely when in 2024 the salt targets were supposed to have been met. Was it January 1? Or was it October 1? This matters for understanding that extent to which the data could be considered "post-implementation" since they were collected in February-May of 2024. I do not need to re-review the manuscript as the editor can verify that this detail was added in the final version.

Thank you for the important point. The government guidance does not provide specific dates for the targets to be met beyond the year. We have made this more explicit in the section of the limitations where we address the issue of when our data collection took place. The sentence that previously read “Our data collection took place predominantly in February/March 2024, so it is possible that we would have seen better adherence to the salt targets if we had collected data later in 2024 (as the targets had to be met in 2024)”, now reads “Our data collection took place predominantly in February/March 2024, so it is possible that we would have seen better adherence to the salt targets if we had collected data later in 2024 (as the targets had to be met in 2024, with no specification of which day or month)” (line 643).

**Other changes to adhere to journal guidelines**

- The formatting of some of the references has been updated to better comply with Vancouver referencing.
- We have corrected one instance where “out-of-home” hadn’t been abbreviated to “OOH” following the first use.
- Information regarding ordering of plots or explanation of plot values has been moved from figure titles to figure captions.
- The numbering of the supplementary files has been shifted down by 1, with the addition of the study protocol as S1 File.

**Corrections**

- Removed word ‘retailers’ at line 562.
