# Supplementary material for: Adherence to voluntary UK sugar, salt, and calorie reduction targets in the highest-grossing restaurant chains: A cross-sectional study
Source: PLoS Med. 2026 May 5;23(5):e1004681. doi: 10.1371/journal.pmed.1004681 (PMC13143115; doi:10.1371/journal.pmed.1004681)
Supplement: Attachment — Submitted filename: Reviewer_Responses_auresp_4.docx [file pmed.1004681.s033.docx]

*In the Abstract:

- Please remove the capitalization of Pizza and Sandwich and we would suggest removing the final part of the sentence "E.g. 76% of...Nero"

This has been changed.

- Please add the number of highest grossing restaurants to the last sentence of the Background sub-section

This has been changed.

- Please revise the wording in the Abstract and main text to say that the menu items at restaurants x,y,z had the lowest adherence to targets rather than the restaurants themselves, e.g., "Papa John's menu items had the lowest adherence" rather than "Papa John's had the lowest..."

This has been changed throughout the paper.

* Please revise the limitations in the Methods and Findings subsection of the Abstract and in the Author summary to focus on methodological limitations, and remove the reference to "mandatory reporting... ". This point about reporting can instead be included and expanded in the the discussion.

The limitation in the abstract now does not refer to the mandatory reporting, and reads “We were unable to account for heterogeneity in item-level sales due to the lack of accessible sales data from the out-of-home food sector, and therefore we could only assess performance against the targets for available items as supposed to purchased items.”

The same change was applied to the author summary which now reads “We were unable to account for the fact that menu items may differ in their sales, and therefore our findings illustrate the adherence of available menu items, as supposed to purchased menu items, against the sugar, salt, and calorie targets.”

* In the main text, please replace the section heading Background with Introduction.

This has been changed.

* Please clarify whether the UK requires or recommends meeting the targets. Targets are referred to as voluntary in the Introduction of the main text but in Background of the Abstract the wording is 'had to be met by...' which suggests these were required and not voluntary.

We have changed ‘had’ to ‘were intended’.

*We would suggest changing this sentence "with some of the highest-grossing companies including McDonald’s, KFC, Domino’s, and Gregg" removing the individual restaurant names and linking to a table identifying all 21 restaurants.

This has been changed.

Figures: Please explain the meaning of the different colours in the figure legends, for example in Figure 1 define the meaning of blue and red.

Figure 1 caption now includes “Main meals are coloured in blue, sides/extras are coloured in red, and desserts are coloured in purple.”.

Figure 2 and 3 captions now includes “Main meal subcategories are coloured in blue, Side categories are coloured in red, and Desserts are coloured in purple.”.

Figure 4 and 5 captions now includes “Burger restaurants are coloured in purple, Chicken restaurants in yellow, Pizza restaurants in Blue, Other Main restaurants in green, and Sandwich restaurants in pink.”.

Figure 6 caption now includes “The dark orange bars indicate the proportion of menu items meeting the target, and the light orange bars indicate the proportion of menu items not meeting the target.”.

Figure 7 caption now includes “The dark blue bars indicate the proportion of menu items meeting the target, and the light blue bars indicate the proportion of menu items not meeting the target.”.

Other changes:

- Rephrasing of this sentence in the discussion for clarity, from “It is possible that healthier menu items are responsible for a smaller proportion of sales” to “It is possible that healthier menu items (items that did meet their sugar, salt, and calorie targets) are responsible for a smaller proportion of sales”.
- In discussion, reference to S2 Table is corrected to S3 Table.
