# Supplementary material for: Adherence to voluntary UK sugar, salt, and calorie reduction targets in the highest-grossing restaurant chains: A cross-sectional study
Source: PLoS Med. 2026 May 5;23(5):e1004681. doi: 10.1371/journal.pmed.1004681 (PMC13143115; doi:10.1371/journal.pmed.1004681)
Supplement: Attachment — Submitted filename: Reviewer Comment Responses.docx [file pmed.1004681.s031.docx]

-----------------------------------------------------------

Comments from the academic editor:

A suggestion for a more appropriate title would be: Voluntary self-regulation of restaurant quality does not work: an evaluation of the UK restaurant nutrient guidelines.

Thank you for the suggestion. We personally believe that drawing the conclusion that voluntary regulation of restaurant quality does not work is too strong of a conclusion to draw from a single study. We also believe that including the specific name of the Government scheme, being the ‘UK Government’s sugar, salt, and calorie reduction targets’, will aid literature searches that are looking for evidence on the effectiveness of this particular policy, rather than ‘restaurant nutrient guidelines’ which could refer to nutrition guidelines more broadly. However, we do agree that including ‘voluntary’ in the title is important, so we have chosen the new title to be as follows: “Voluntary UK sugar, salt, and calorie reduction targets: A cross-sectional study assessing adherence within the highest-grossing restaurants”.

-----------------------------------------------------------

Comments from the reviewers:

Reviewer #1:

MAJOR COMMENTS

M1. My main statistical issue with the manuscript is perhaps a bit of a subtle point: inferential statistics are actually not technically applicable here as the data included in the study are not a random sample of restaurants and menu items but are an exhaustive list (with several acknowledged non-random exclusions) of menu items from the largest 21 chain restaurants. This means that where there is a difference in nutritional values or in adherence proportion, this is very much true and not subject to sampling variation (i.e. the sample means reported here are the actual population means for the top 21 restaurants included). As such, p-values are not technically meaningful here.

Thank you for your insight. After further consultation with a statistician, we have decided to remove all inferential statistics and stick to a descriptive interpretation of the results. We agree with your point that our data does not consist of a random sample of menu items, and therefore we can conclude that the differences seen in nutrient content and target adherence between restaurants/subcategories are the actual differences, and not a result of sampling variation. However, it could be argued that our sample is a random selection within time, and therefore inferential statistics would be warranted if we wanted to make generalisations in nutrient content/target adherence beyond the specific time point where our data was collected. With this in mind, we have opted to remove all inferential statistics but make changes to wording, to make it clear that we are only making inferences about restaurants’/subcategories’ nutrient content/target adherence for the specific time point at which we collected the data. Following this, line 519 now reads “This study shows that only 43% of menu items from the highest-grossing UK restaurant chains had met all of their reduction targets at the start of 2024”.

M2. Setting aside the more fundamental issue from comment M1, the logistic regression analyses described on l.271-283 and associated results on Table S20 and l.542-562 are not technically incorrect, but they are not the best analysis here and do not directly answer the research question ("Does adherence differ by restaurant / restaurant type?") that, I understood, the authors wanted to answer. Similar to the Kruskal-Wallis/Dunn test procedure for comparing levels of salt/sugar/calories, an omnibus test whether adherence to targets differed by restaurant or restaurant type would be more directly meaningful. An obvious candidate for this would be Fisher's exact test (or the chi-squared test if expected cell counts are large enough) for adherence and restaurant (resp. restaurant type). An alternative could be to stick with the logistic regression model, but then do a likelihood ratio test comparing the model with the restaurant dummy variables included to the null model with only an intercept term. Logistic regression would allow accounting for confounders, but that was not done anyway (and likely irrelevant here anyway), so there is not really any need for this approach. Currently the logistic regression procedure depends on choosing a reference restaurant (resp. restaurant type) and all the p-values are just whether each other restaurant (resp. restaurant type) differs from the (arbitrarily) chosen reference restaurant (resp. restaurant type) - I do not think that this is not quite what the authors set out to do. If the authors decide on taking onboard my advice for an omnibus test, then pairwise post-hoc comparisons could still be made with that approach, though I am less convinced how much value this would add beyond the descriptive summaries of the adherence results already given on Figures 6 and 7 and l.501-541.

As we have removed all inferential statistics, this comment no longer applies.

M3. Sales-weighting would be key. While acknowledged by the authors as a limitation of the study, this is really key. Perhaps some simple online survey could be done to get some, perhaps imperfect, data on menu item popularity, that could be used for a sensitivity analysis? To illustrate the scale of the issue, e.g. from Figure 1 it is clear that McDonald has more breakfast items than burger items on their menu, but I am fairly certain they sell many more burgers than breakfasts...

We entirely agree that the lack of sales weighting is a major limitation of the study, which we note from line 588. However, we do not feel that we would gain sufficient additional insight from conducting what would need to be a large online survey of self-reported product purchasing habits that would represent a different timepoint (with different products potentially now available). To highlight how our findings are still useful in their current form, we describe how our findings show that companies should be able to improve nutritional quality without changing the types of foods they offer (line 582). We also highlight how the introduction of mandatory reporting for healthy sales as outlined in the NHS 10 year plan, could address the problem of lacking sales data (line 638). We note how the government’s salt reduction target progress report was unable to do a sales-weighted analysis for OOH due to the lack of sales data (line 593), which further emphasises how limiting the lack of available sales data is for monitoring progress towards these targets.

SPECIFIC POINTS

S1. STROBE compliance / Table S1:

- Item 3: The objectives of the study should be more clearly stated as is currently the case. Given the focus in results on not just adherence, but also nutritional values directly, the message of the paper was not as clear as it could have been.

The aim of the study stated in the Background section (line 170) now refers to assessing nutrient content as well as target adherence, reading as “This study aimed to assess the nutritional content and adherence to the UK Government’s sugar, salt, and calorie reduction targets, for food items offered by the highest grossing restaurant chains in the UK, in 2024.”. This new aim has also been updated in the Objectives box of STROBE table S1.

- Item 9: Bias needs to be more discussed as there are a number of design and analysis choices that directly bear on this, such as the inclusion of the largest 21 restaurants rather than a perhaps a more systematic approach resulting in a larger or smaller number meeting specific eligibility criteria, the lack of sales-weighting which could bias results to more exotic menu items etc.

We agree with both of your suggested points, and have added this explanation to the Bias item of the STROBE table: “We acknowledge that bias may be resulting from our selection of the restaurants, as we are only focusing on the highest-grossing restaurant chains, and therefore our findings may not be representative of independent restaurants or smaller chains. In addition, the lack of sales weighting in our findings means that our assessment of a restaurant’s target adherence may not be representative of the menu items that are actually being purchased by customers.”

- Item 12 d: This should probably not have been marked as 'N/A' as essentially there is no random sampling here, but an exhaustive inclusion of all menu items from the top 21 largest chain restaurants; see comment M1. So this could have been acknowledged in this section.

We agree that this distinction is important, and therefore have included the following sentence in place of N/A: “This study analysed nutritional data for all food items offered by the highest-grossing restaurant chains in the UK, and therefore our analysis was based on the complete population of menu items from these restaurants, as supposed to a randomly selected sample. Considering this, we opted for a descriptive, rather than inferential analysis approach”

S2. The selection of the top 21 largest restaurant chains is a bit puzzling to me. Either a random sample from a reasonably exhaustive list of restaurants or a more systematic approach including all restaurants meeting a pre-specified set of eligibility criteria would seem a better approach to me. The reasons for including only the top 21 largest restaurants is not very clearly explained in the manuscript other than that 20 would have been a nice round number (the reason why 1 more restaurant was included in the end is explained very clearly though).

In the Background section, we explain how the out-of-home food sector is dominated by a small number of multinational companies that make billions of pounds in revenue each year, demonstrating the significant impact that these companies have over diet and health (line 145). We agree that whilst we refer to this in the Background section, we do not make it clear in the Methods section that this is the reason why we chose to only focus on the highest-grossing restaurant chains, so we have added this sentence to the beginning of the ‘Identifying Restaurants’ section of the Methods: “This study focuses on the highest-grossing restaurant chains in the UK, as these restaurants have the largest impact on diets within the OOH food sector.” (line 185)

S3. The imputation strategy described on l.236-237 is in general sound, but as a single-value imputation it will introduce some bias and also underestimate uncertainty of the unknown serving size. A slightly improved version would be to fit a distribution to the sub-category serving sizes, then impute randomly from that distribution multiple times for each missing serving size, analyse the data for each imputed version of the dataset, then pool results using Rubin's rules. I acknowledge that this may be overkill here, however - unclear how much missing data there were and how wide the distributions of serving sizes per sub-category were.

Thank you for the recommendation. Following our consultation with a statistician regarding removing the inferential statistics (comment M1), we discussed that the suggested imputation strategy would only be necessary if we were to include inferential statistics, as the purpose of it would be to narrow the confidence intervals in our analyses. As we have removed inferential statistics, we opted instead to do a sensitivity analysis where instead of using the subcategory average serving size as our imputation value, we used the lower quartile and upper quartile, to observe how robust our findings were to changing the imputation approach. We have added a description of this sensitivity analysis in the ‘Sensitivity Analyses’ section of the methods (line 310), and have noted it as a deviation from protocol in the ‘Deviations from Protocol’ section (line 353). The findings of the sensitivity analysis are described from line 500.

S4. I am slightly sceptical of the value of the comparisons between restaurants and sub-categories described on l.241-261 and results presented on l.411-429. I would expect these to differ given that different restaurants will focus on different styles of food (some healthier than others) and clearly you would expect food sub-categories to differ anyway. The pairwise, post-hoc tests are even less meaningful in my opinion, especially as the choice to include the largest 21 restaurants is a bit arbitrary (see comment S2). Similarly, for the results on l.358-360, I do not think any of these means across sub-categories are meaningful - this is almost literally mixing apples and bananas (or sauces and chicken if you will). For example, the sauce mean values per serving are of course going to be much lower as a sauce serving is just a lot less than a burger serving. Likewise, e.g. desserts will of course be more sugary than mains and sauces, meant to give flavour, will of course be high on salt. So to some extent these comparisons are a bit trivial. I would focus on just a descriptive analysis of the nutrient values by restaurant and sub-category. This would also, I think keep a clearer focus on adherence to the government target values which is the main objective of the study. The Discussion section is firmly only on adherence results, and does not discuss the results from the statistical comparisons of nutritional values, which also suggests that there is no need for these analyses.

We believe that the removal of inferential statistics has addressed this comment.

S5. Another key limitation, in my view, is the reliance on restaurant-reported data. This has, however, been acknowledged as a limitation by the authors on l.606-611, so I think that is fine.

Thank you.

S6. Language, particularly in the introduction / background section and in parts of the discussion, is a bit confusing as the authors repeatedly mention REDUCTION / CHANGE in nutritional values. But of course with a single cross-sectional timepoint, it is not possible to look at changes, but only overall levels and adherence to current government targets. This becomes quite clear in Methods and Results, but I think the authors may consider revising the introductory and discussion text. The reduction / change is the aim of the government targets, but what the study assesses is not reduction / change, but current compliance.

We appreciate the reviewer picking up on this point, and agree it is an important distinction. We have edited our phrasing in the following places to reflect this:

- Background section of the Abstract (line 42): “There is limited evidence for how the out-of-home sector is performing against these targets…”.
- Comparison to Other Literature section of Discussion (line 535): “To our knowledge, only two studies have looked at company-level performance against the reduction targets.”
- Comparison to Other Literature section of Discussion (line 545): “Pepper et al (20) found that 4/48 OOH companies met the 20% reduction target for Desserts in 2020…”

We feel as though our description of the findings from the Government progress reports in the Background section can be kept as currently phrased as these reports did measure change/reduction directly, and we use it more to demonstrate how the reduction targets appear to not have been successful so far (and less successful in OOH than grocery and manufacturing sector).

MINOR CLARIFICATIONS AND TYPOS

CT1. L.195: This sentence should start with "Second, [...]", given the prior use of "first, [...]" on l.188.

This has been updated.

CT2. L.201-203: Unclear to me what the authors mean here.

We agree that this explanation is not clear and have updated the phrasing to be as follows: “To align with the guidance that an item should not have both a calorie and a sugar target, we first categorised menu items by the sugar target categories, and then categorised the remaining items by the calorie target categories, as the sugar reduction programme preceded the calorie reduction programme.”

CT3. L.208-212: This is a bit unclear as it seems to mix up both restaurant and item categorisations?

We can understand how this is not clear – the restaurant types were based on the predominant main meal subcategory of food that the restaurant offered. We have updated the phrasing to better reflect this (line 251): “We divided our 21 restaurants into the following five ‘restaurant types’, based on the predominant main meal subcategory that the restaurant offered: Burger restaurants, Chicken restaurants, Pizza restaurants, Sandwich restaurants, and Other Mains restaurants.”.

CT4. L.215: RStudio is just an interface to the underlying statistical computational environment R. The version number stated is an R version number not an R Studio version number. R should be cited properly as per R guidance, as should all of the packages that are listed -->type 'citation()' at the console for how to cite R and 'citation("dplyr")', for example, for how to cite a package (package 'dplyr' in the example).

Thank you for the advice, we have now cited R instead of RStudio, and have referenced R and all of the listed packages.

CT5. L.256-261: This is generally statistically sound (with the caveat of my general comment M1), but can the authors clarify that Dunn's tests were only done when the overall KW test was statistically significant?

This comment no longer applies as we have removed inferential statistics.

CT6. L.292: While both singular and plural are regularly used, data is more commonly (and more correctly in my opinion) used in the plural form.

This comment no longer applies as we have removed this paragraph (due to removing inferential statistics)

CT7. L. 291-292: Please note that if sample size is large enough, there would not really be a problem with ANOVA even if the data are not normally distributed (ANOVA requires the sample means not the data to be normally distributed and due to the Central Limit Theorem, sample means will be approximately normally distributed regardless of the individual data distributions as long as the sample size is large enough; how large depends on the extent of the non-normality of the data...). I do not expect the authors to change anything in response to this comment, but just wanted to clarify that as currently stated, the reason for not doing ANOVA is really only a reason if sample sizes per level of the grouping variable are small/moderate and the non-normality is severe.

Thank you for your insight. As we have removed inferential statistics, this comment is no longer applicable.

CT8. l.330: Unclear what NPM is.

We apologise for the oversight and have now changed this to the complete name (line 360): “The planned analysis using the UK Ofcom/FSA Nutrient Profile Model as an additional assessment of nutritional quality, will be presented in a separate paper.”

CT9. Figure 1: Maybe a software restriction, but I don't think there is a need in the Desserts category to have both a total and a 'Desserts' row as they are the same (and also the heat map colour coding is irrelevant here as there is a single sub-category).

Thank you for the suggestion, this has been changed.

CT10. For the percentages given for the adherence results, it would be helpful if frequencies are stated as well, i.e. "x% (n/N)".

We agree this would add clarity, so we have done this throughout the reporting of the adherence results (beginning line 446).

CT11. Figures 6 and 7 are very nice. In addition to the total items / restaurant stated in brackets at the end of each row, I would also add the actual count near the % figure. I would also move the note on l.520-522 of the main text into the captions for each of these two figures - that would be much clearer.

Unfortunately we were unable to add the count to the plots in a way that was clear to read, whilst adhering to the journal guidelines for figure dimensions. We have moved the note to the figure caption.

Reviewer 2:

ABSTRACT

Line 38: It would be helpful to know in what year the UK gov set these targets for restaurants, or the year by which restaurants were supposed to meet them. I see this info is partially in the discussion but should at least be in the introduction to provide important context (and, if possible, fit into the abstract). Also, if the salt targets are not supposed to be met until 2025 ("2024/5" in line 687), and these data were collected in 2024, that is important for the reader to know upfront. It should also be acknowledged as a major limitation.

We agree this is an important point. We have added this sentence to the abstract “The sugar targets had to be met in 2020, the salt targets in 2024, and the calorie targets in 2025 (extended from 2024 due to Covid-19).”. In the Background section, we had already provided the years for which the sugar and calorie targets had to be met, but we have now added this explicitly for the salt reduction targets (line 114): “The first reduction and reformulation targets for industry were published in 2006 and have since been revised on several occasions, with the most recent revision in 2020 (for targets to be met by 2024)…”. We have also added a paragraph to the strengths and limitations section to address this point (beginning line 617).

Line 41: "Progressing" implies this study will look at change, but it is cross-sectional.

We agree this phrasing isn’t accurate and have changed it to the following: “There is limited evidence for how the out-of-home sector is performing against these targets”.

Line 68 (and 578 and elsewhere): "Engagement" is not quite the right word. That is not what this study is measuring. There may have been a large increase in adherence or none. Change in adherence, which this study did not measure, would be an appropriate proxy for engagement. But because this study did not measure change, a different word is recommended. One possible suggestion is "adherence to targets."

We agree the term ‘engagement’ could be misleading, so we have followed your suggestion of replacing all instances of ‘engagement’ with ‘adherence to targets’ (line 42).

INTRODUCTION

Line 80: Are any of these mandatory?

This sentence now reads (line 108): “68 countries have a salt reduction policy in place (4), and some countries such as the UK, Brazil, New Zealand, and USA, have further initiatives for sugar reduction (5), with the majority of such schemes being voluntary (4,5).” To highlight how there are some mandatory schemes but the majority are voluntary.

Line 85: What was salt intake on average in 2004 when the program was established? In other words, what % reduction was the program trying to achieve?

I have changed the wording to clarify this (line 114): “following advice from the Scientific Advisory Committee on Nutrition to reduce the population average salt intake by approximately 10%, to 6g a day.”

Line 88: What is an example of one of 24 OOH category-specific targets?

I have added an example of a category for both the grocery and OOH targets (line 117): “covering 84 category-specific salt targets for grocery foods (e.g., Ready Meals), and 24 for the out-of-home (OOH) sector (e.g., Seasoned Fries)”.

Line 109: Precisely, what percentage of energy or weekly meals comes from the OOH sector?

We have followed up the previous sentence with a finding from the UK Diet and Nutrition Survey which is more up to date, and quantifies the contribution of OOH foods to diets: “The UK Diet and Nutrition Survey finds 72% of respondents reported purchasing food or drink from an OOH establishment in the past week, with the largest proportion of these consumers being children aged 11 to 18 years old.” (line 140)

METHODS

Line 157: Suggest "location" instead of "site" as "site" may be misconstrued to refer to website. Also, how many were excluded because of this?

We agree this is unclear, and have followed your suggestion of using “location” instead. (line 194)

Line 175: How did the authors deal with combo/value meals? For example, for fries offered by itself AND as part of a combo, were they counted as just one item or twice?

Thank you for picking that up, we have now clarified that (line 213): “Meals that combined several food items that could be purchased separately, were also included as their own menu item.”

Lines 236-9: For what % of items did you need to impute serving size or weight?

We agree that this is an important point to include – we have now added a sentence to clarify this (line 368), and have added supplementary tables S5 and S6 to provide more detail on the affected products.

Line 330: NPM? Nutrition Profile Model?

Yes- we have now written this out in full to be more clear.

DISCUSSION

Usually, the strengths and limitations section goes just before the conclusion and after the discussion of literature.

We have moved the ‘Comparison to Other Literature’ section to be before the ‘Strengths and Limitations’ section, but because some of the study’s limitations feed into the policy implications, we have opted to keep the ‘Policy Implications’ section last.

Line 604: If the authors agree, they could recommend additional sales-weighted targets and mandatory reporting to the government

The government guidance already recommends that the targets should be applied as sales-weighted, so we have added this sentence (line 591) to clarify “The technical guidance for the reduction targets highlights that applying the targets based on sales-weighted averages is the gold standard approach, however this is not always possible, particularly for the OOH sector.”. We agree with your suggestion of mandatory reporting to the government, and have now added reference to the NHS 10 year plan to support this (line 638).

Line 676: Can the authors provide a few specific examples of mandatory OOH regulations?

We have included examples in brackets so the sentence now reads (line 634): “with research from other countries evidencing the increased effectiveness of mandatory (e.g., maximum limits or required declaration of salt content) versus voluntary (e.g., suggested limit on salt content) nutrition policies in inciting reformulation”

Line 683: Again, "make progress" suggests this study examined change, but it did not. Please rephrase.

We have changed “make progress towards” to “meet”.

To be addressed in the introduction and/or discussion: How do the methods employed herein differ from those used in other studies of OOH and nutrient changes or adherence to targets? Although this is a cross-sectional study, are there existing studies from years ago that the authors could use as a rough "baseline" to discuss changes? Or are the methods too different from those employed here?

The two studies that we have referred to in the Background and Discussion (Bandy et al and Pepper et al) are the only two papers we are aware of that have directly measured adherence against the reduction targets. The majority of the literature has either measured changes over time in nutrient content for food categories that were included by the reduction targets, or measured changes in population intake over time, and attributed this change to be a result of the reduction programmes. Therefore, there are unfortunately no existing studies from years ago to use as a baseline, so we are only able to draw conclusions about target adherence in 2024.

*Other changes made outside of the reviewer’s suggestions:*

1) *Updating the subcategory averages*

*We noticed that some of the subcategory averages that had been used to replace missing serving sizes in the primary analysis, were based on a previous version of the dataset, where some menu items had since been re-categorised and hence the subcategory average had changed. The table below shows the old versus new subcategory average serving sizes.*

| Subcategory | Old average serving size | New average serving size | Absolute difference between old and new serving size |
| --- | --- | --- | --- |
| Pizzas | 203.79 | 229.50 | 25.71 |
| Other Mains | 470.86 | 455.62 | 15.24 |
| Breakfast Items | 168.90 | 167.48 | 1.42 |
| Sandwiches | 190.43 | 191.24 | 0.81 |
| Desserts | 77.93 | 78.14 | 0.21 |
| Other Sides | 114.33 | 114.29 | 0.04 |
| Potato Sides | 111.83 | 111.83 | 0.00 |
| Salads | 219.54 | 219.54 | 0.00 |
| Children's | 197.75 | 197.75 | 0.00 |
| Sauces | 30.54 | 30.54 | 0.00 |
| Chicken | 243.57 | 243.57 | 0.00 |
| Burgers | 239.39 | 239.39 | 0.00 |

*The primary analysis results have been updated where missing serving sizes have been replaced by subcategory averages from the current dataset. This had a very minimal effect on the results – the proportion of menu items meeting sugar, salt, calorie and all applicable targets across all restaurants stayed the same, with only the following differences seen at the subcategory/restaurant level:*

- *‘Other Mains’ all applicable target adherence dropped 1% (40 to 39%).*
- *‘Other Mains’ salt target adherence dropped 1% (46 to 45%).*
- *Vintage Inns’ all applicable target adherence dropped 2% (42 to 40%).*
- *Vintage Inns’ salt target adherence dropped 2% (60 to 58%).*
- *Pizza Hut’s salt target adherence increased 1% (67 to 68%).*

*There were very minimal changes to overall mean nutrient content per 100g and per serving. There were small changes at the subcategory/restaurant level, with the largest difference seen for kcal per 100g for the Pizza subcategory (average 313.24kcal to now 293.89kcal) and Pizza Hut restaurant (average 334.64kcal to now 302.70kcal), but it can be seen above that this had very little impact on the target adherence results.*

*The corrected version of the dataset has been uploaded to the Oxford University Research Archive.*

2) *NHS 10 Year Plan*

*We have added a reference to the NHS 10 Year Plan in the introduction (line 167) and discussion (626), as the findings of our study provide support for two key suggestions made in the plan – mandatory healthy sales reporting, and mandatory targets for healthy sales.*

3) *Salt Target Categorisation (line 242)*

*We have added extra clarification to how the salt targets were applied at line 242.*

4) *Added missing reference (ref 40).*
